# Selective pharmacological inhibition of DDR1 prevents experimentally-induced glomerulonephritis in prevention and therapeutic regime

**DOI:** 10.1186/s12967-018-1524-5

**Published:** 2018-06-01

**Authors:** Solange Moll, Yukari Yasui, Ahmed Abed, Takeshi Murata, Hideaki Shimada, Akira Maeda, Naoshi Fukushima, Masakazu Kanamori, Sabine Uhles, Laura Badi, Thomas Cagarelli, Ivan Formentini, Faye Drawnel, Guy Georges, Tobias Bergauer, Rodolfo Gasser, R. Daniel Bonfil, Rafael Fridman, Hans Richter, Juergen Funk, Marcus J. Moeller, Christos Chatziantoniou, Marco Prunotto

**Affiliations:** 1 Department of Pathology, University Hospital of Geneva, Geneva, Switzerland; 2 Research Division, Chugai Pharmaceutical Co., Ltd, Tokyo, Japan; 3 INSERM, UMR S 1155, Hôpital Tenon, 75020 Paris, France; 4 Roche Pharma Research and Early Development, Roche Innovation Center Basel, Basel, Switzerland; 5 Roche Pharma Research and Early Development, Roche Innovation Center Munich, Munich, Germany; 6 Department of Pathology, College of Medical Sciences, Nova Southeastern University, Fort Lauderdale, FL USA; 7 Department of Pathology, Wayne State University, Detroit, MI USA; 8 Department of Nephrology and Clinical Immunology, RWTH University, Aachen, Germany; 9 Present Address: Chugai Pharmabody Research Pte. Ltd., Singapore, Singapore; 10 Present Address: Late Stage, AstraZeneca, Göteborgs, Sweden; 11 Present Address: Office of Innovation, Immunology, Infectious Diseases & Ophthalmology (I2O), Roche and Genentech Late Stage Development, 124 Grenzacherstrasse, 4070 Basel, Switzerland; 12 School of Pharmaceutical Sciences, University of Geneva, Geneva, Switzerland

**Keywords:** Fibrosis, DDR1 inhibition, Glomerulosclerosis, CKD

## Abstract

**Background:**

Discoidin domain receptor 1 (DDR1) is a collagen-activated receptor tyrosine kinase extensively implicated in diseases such as cancer, atherosclerosis and fibrosis. Multiple preclinical studies, performed using either a gene deletion or a gene silencing approaches, have shown this receptor being a major driver target of fibrosis and glomerulosclerosis.

**Methods:**

The present study investigated the role and relevance of DDR1 in human crescentic glomerulonephritis (GN). Detailed DDR1 expression was first characterized in detail in human GN biopsies using a novel selective anti-DDR1 antibody using immunohistochemistry. Subsequently the protective role of DDR1 was investigated using a highly selective, novel, small molecule inhibitor in a nephrotoxic serum (NTS) GN model in a prophylactic regime and in the NEP25 GN mouse model using a therapeutic intervention regime.

**Results:**

DDR1 expression was shown to be mainly limited to renal epithelium. In humans, DDR1 is highly induced in injured podocytes, in bridging cells expressing both parietal epithelial cell (PEC) and podocyte markers and in a subset of PECs forming the cellular crescents in human GN. Pharmacological inhibition of DDR1 in NTS improved both renal function and histological parameters. These results, obtained using a prophylactic regime, were confirmed in the NEP25 GN mouse model using a therapeutic intervention regime. Gene expression analysis of NTS showed that pharmacological blockade of DDR1 specifically reverted fibrotic and inflammatory gene networks and modulated expression of the glomerular cell gene signature, further validating DDR1 as a major mediator of cell fate in podocytes and PECs.

**Conclusions:**

Together, these results suggest that DDR1 inhibition might be an attractive and promising pharmacological intervention for the treatment of GN, predominantly by targeting the renal epithelium.

**Electronic supplementary material:**

The online version of this article (10.1186/s12967-018-1524-5) contains supplementary material, which is available to authorized users.

## Background

Discoidin domain receptor 1 (DDR1) is a collagen-activated receptor tyrosine kinase (RTK) [1] extensively implicated in diseases such as cancer [2], atherosclerosis [3] and fibrosis [3–5]. In humans, single nucleotide polymorphisms of *DDR1* are associated with susceptibility for and disease progression of childhood IgA nephropathy [6]. In multiple preclinical studies DDR1 has been shown to play a major role in the pathogenesis of fibrosis and glomerulosclerosis [7–13]. The evidence for a protective role of DDR1 in glomerulosclerosis has been supported so far by gene knockout (KO) experiments or the use of antisense oligonucleotides (ASO), with both technologies relying on reduction of total DDR1 protein [1–5]. Although these techniques can demonstrate mechanism, they have very limited translational potential. Knock-out mouse models mimic prophylactic regimens, which are not applicable to patients suffering from a substantial loss of kidney function. ASOs, though tested in a therapeutic intervention regimen by one of the co-authors [10], are predominantly cleared by the liver and kidneys, and thus represent a non-preferred clinical scenario in renal-impaired patients.

The present study includes results originally generated as part of a pharmaceutical program, aimed at the creation of a safe and effective DDR1 inhibitor to be used in patients affected by glomerulonephritis (GN) [14]. Firstly, we show in depth characterisation of DDR1 expression in normal human kidney and in renal biopsies from patients with crescentic GN, using a newly-developed highly specific anti-DDR1 antibody. These translational data were essential to further enhance confidence in the initiation and progression of a medicinal chemistry effort to generate an exquisitely selective and potent DDR1 inhibitor (DDR1i). The characterisation of DDR1i in two different mouse models of GN [14], in both prophylactic and therapeutic regimens, is presented here. Gene expression profiles of selective DDR1i-treated animals were also profiled in order to gain further knowledge regarding the pathways and networks selectively modulated by drug targeting. The data suggest that DDR1 is an important player in human GN and that its pharmacological inhibition is translatable into a valid therapeutic intervention tested in preclinical GN models.

## Results

### DDR1 is exclusively expressed in renal epithelium under physiological conditions

**Fig. 1:**
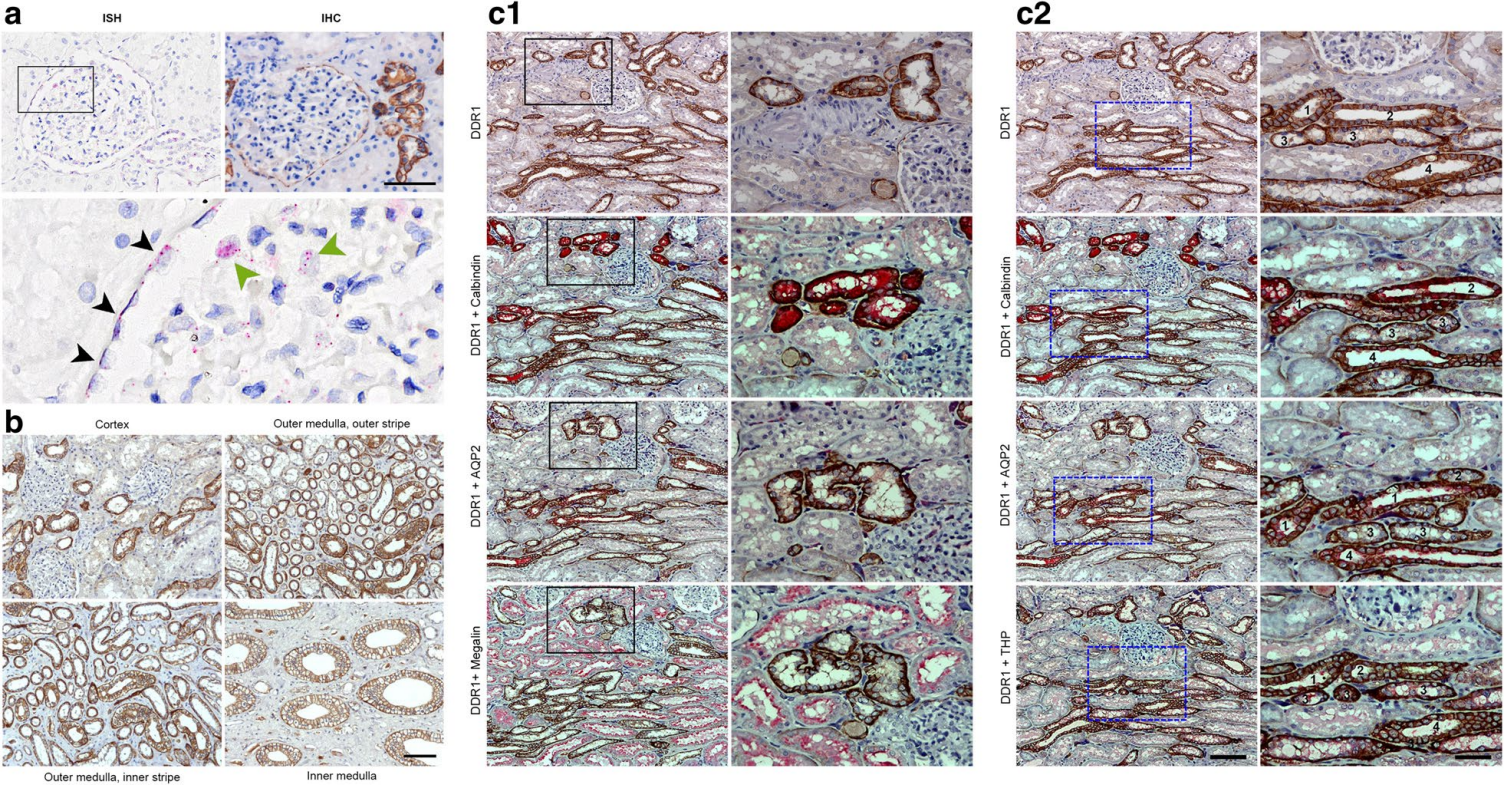
Localisation of DDR1 in human control kidney. **a** DDR1 in situ hybridization (ISH) and immunohistochemistry (IHC) analyses in normal human kidney. (Scale bar = 100 μm). ISH higher magnification rectangle: DDR1 positive podocyte (black arrowheads), DDR1 positive podocytes (green arrowheads). **b** DDR1 immunostaining in the nomal human kidney with representative images of the cortex, outer medulla (outer and inner stripes) and inner medulla. Note the DDR1 membranous staining of the tubules. (Magnification ×125, scale bar 50 μm). **c** Serial sections of normal human kidney were double immunostained with DDR1 and specific tubular antibodies against Megalin, Calbindin, Tamm–Horsfall Protein (THP or Uromodulin) or Aquaporin 2 (AQP2). **c1** Representative micrographs of the cortex show DDR1 protein localization in the distal convoluted tubules (Calbindin+, Aquaporin 2−, Megalin−). Proximal tubules (Megalin+) are DDR1 negatively stained. Boxed areas are enlarged in the right side of the figure. **c2** Representative micrographs of the medullar rays of the cortex show DDR1 protein localization in the connecting tubules (1: Calbindin+, Aquaporin 2+, THP−), distal convoluted tubules (2: Calbindin+, Aquaporin 2−, THP−), thick ascending limbs of the Henle’s loop/pars recta of the distal tubules (3: THP+, Calbindin−, Aquaporin 2−) and collecting ducts (4: Aquaporin 2+, Calbindin−, THP−). Boxed areas are enlarged in the right side of the figure. Magnification ×100, scale bar = 100 μm; Boxed areas Magnification ×250, scale bar = 50 μm

DDR1 mRNA and protein expression is restricted to the glomerular parietal epithelial cells (PECs) of the Bowman’s capsule and to podocytes and some tubules (Fig. 1a). Immunohistochemistry (IHC) failed to reveal the subtle podocyte staining detected with ISH, probably due to differential detection thresholds, showed a characteristic membranous localization of DDR1. DDR1 immunostaining was observed in the tubules of the cortex and of both outer and inner medulla, most likely in the distal nephron (Fig. 1b). Double immunostaining with specific tubular markers (Megalin, Calbindin, Tamm–Horsfall Protein/Uromodulin and Aquaporin 2) on serial sections confirmed DDR1 protein localization in the distal parts of the tubule (distal convoluted tubule, thick ascending limb of the Henle’s loop/pars recta distal tubule, connecting tubule and collecting duct) (Fig. 1c). No DDR1 staining was detected in the interstitial space or in vessels, using both ISH and IHC techniques. Taken together, these data demonstrate that DDR1 expression in the normal human kidney is exclusively limited to renal epithelial cells, PECs and podocytes in the glomerulus and tubular cells in the distal part of the nephron.

### DDR1 protein is abnormally induced during crescentic glomerulonephritis in humans and is detected in cellular crescents

**Fig. 2:**
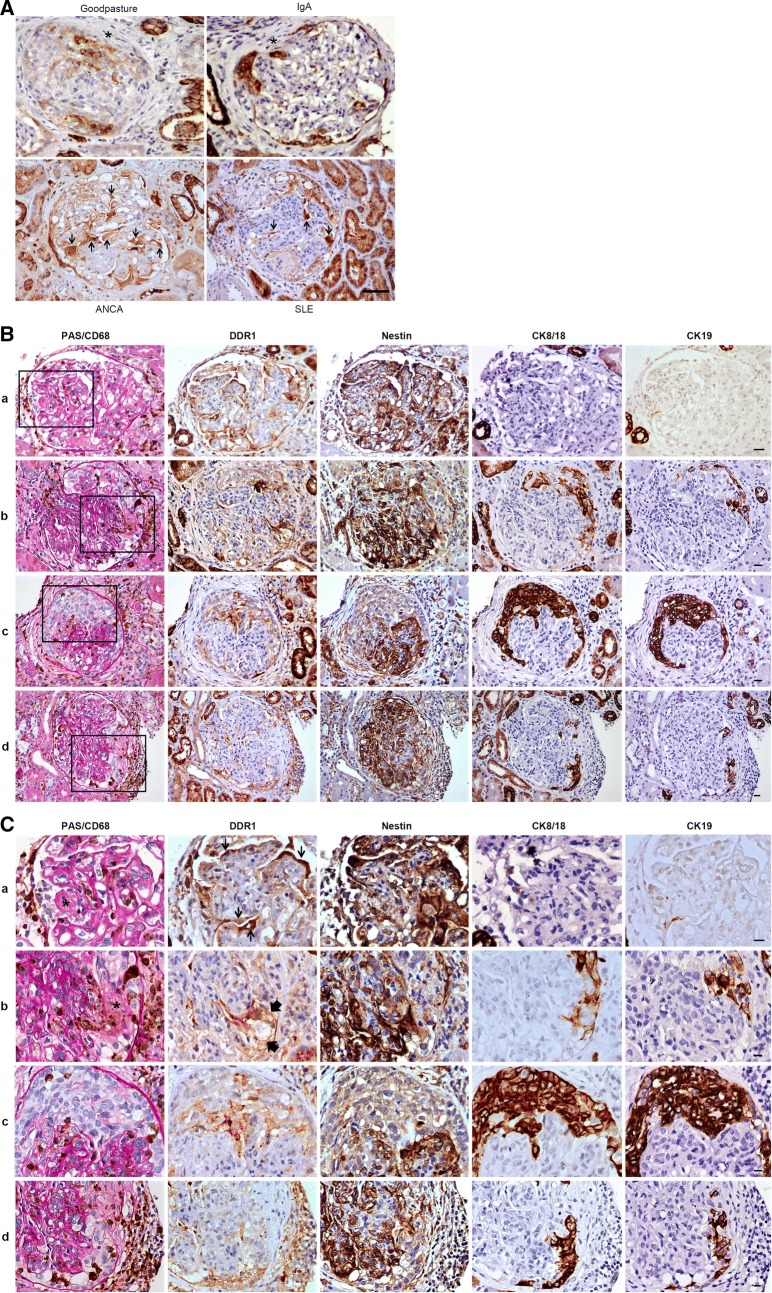
DDR1 is highly induced in crescentic glomerulonephritis. **A** DDR1 expression in 4 different types of human crescentic glomerulonephritis. DDR1 immunostaining in human kidney biopsies from patients with 4 different types of crescentic glomerulonephritis (GN): Goodpasture’s syndrome (patient #1), ANCA-associated GN (patient #12), IgA GN (patient #19) and lupus GN class IV-G (A/C) (patient #29). DDR1 is expressed in the cellular crescents of all 4 types of crescentic GN with variability in the staining intensity and expression limited within crescents to a subset of cells. Injured podocytes and bridging podocytes (arrows) strongly express DDR1. Fibrous crescent (*) are DDR1 negative. Magnification ×200 for SLE, ×250 for ANCA, ×320 for Goopasture and IgA, Scale bar = 20 μm. **B**–**C** Detailed analysis of DDR1 expresion during crescent formation. Serial sections of human kidney biopsies from patients with lupus GN class IV-G (A/C) (patients #25 and #29) were immunostained with specific antibodies against CD68/PAS (Periodic Acid Schiff), DDR1, nestin, cytokeratin 8/18 or cytokeratin 19 proteins. **B** Representative micrographs show expression and localization of each protein in the glomeruli. Boxed areas represent crescentic lesion and are enlarged in **C**. Lines a–d illustrate 4 different morphological stages of the crescent formation; a: early stage with fibrin within the glomerular capillary lumen and presence of 2–3 layers of cells in Bowman’s space; b: early stage with gap in the glomerular capillary wall, plasma proteins in Bowman’s space and cellular crescent formation; c: active hypercellular circumferential crescent compressing the glomerular tuft; d: advanced stage with fibrocellular crescent, capsular rupture and periglomerular inflammation. Line a = patient #25; lines b–d = patient #29. Fine arrows = injured podocytes; large arrows = bridging cells; * = fibrin and plasma proteins within the glomerular capillary lumen or in Bowman’s space. Magnification **A** line a: ×320, b and c: ×250, d: ×200; **B** line a: ×640, b and c: ×500 d: ×400; Scale bar = 20 μm

Twenty-nine biopsies with cellular and/or fibrous crescents were obtained from patients with Goodpasture’s syndrome (5 cases), ANCA-associated GN (7 cases), IgA GN (12 cases) and lupus GN (5 cases: class III and IV according to the ISN/RPS classification) (Table 1: 14 males and 15 females, mean age 56 ± 21 years, mean serum creatinine 199 ± 175 μmol/l, mean blood urea nitrogen (BUN) 11 ± 8 mg/dl, mean proteinuria 3 ± 3 g/l). Twenty-five patients had biopsies performed for diagnosis of acute renal insufficiency and 4 for follow-up of previously treated crescentic GN (patients #4, 25, 28 and 29). Glomerulus number per biopsy varied from 7 to 54 (mean 24 ± 15), globally sclerosed glomeruli from 0 to 23 (mean 2 ± 6) and crescents, either cellular or fibrous, from 1 to 30 (mean 9.7 ± 8.7). DDR1 immunostaining was evaluated in each crescentic glomerulus as positive or negative. DDR1 protein was detected in most of the cellular crescents of all four types of crescentic GN (Table 1, Fig. 2A). DDR1 staining intensity was variable between crescents within the same biopsy and expression limited to a subset of cells forming the cellular crescent. In addition to positive cellular crescents, DDR1 immunoreactivity was detected in injured podocytes (Fig. 2A). DDR1^+^ podocytes were observed in most of the glomeruli, with or without crescent lesion. Furthermore, some of these podocytes appeared to adhere to both the glomerular basement membrane and the parietal basement membrane, forming podocyte bridges between the glomerular tuft and the Bowman’s capsule. All fibrous crescents were DDR1 negative. It should also be noted that DDR1 staining was increased in distal tubules and detected in some cases, particularly SLE cases, in both distal and proximal tubules as depicted in Fig. 2A.

**Table 1:** Clinical, biological and histo-pathological data from the 29 patients with crescentic glomerulonephritis and DDR1 immunostaining evaluation of the crescents as positive or negative

Patient nb	Clinical data							Histological data						
	Diagnostic	Age/gender	SCreat (µmol/l)	eGFR ml/min/1.73 m^2^	BUN mg/dl	Proteinuria (g/l)	Hematuria	Glomeruli		Cellular crescents		Fibrous crescents		TIF %
								Total	Globally sclerosed	DDR1+	DDR1−	DDR1+	DDR1−	
1	Goodpasture	81/F	478	8	18.9	15	pos	8	0	5	0	0	0	25
2		85/F	776	4	32.7	3.68	pos	37	23	3	0	0	2	75
3		22/M	113	> 60	5.3	1.54	pos	9	0	3	0	0	0	0
4		21/M	196	38	10	0.64	pos	14	0	7	0	0	2	10
5		72/M	117	98	10	9	pos	42	0	3	0	0	1	0
6	ANCA	82/F	678	15	26.2	2.5	pos	54	20	0	27	0	3	0
7		74/M	312	16	25.3	0.58	pos	32	0	27	1	0	2	35
8		78/F	84	58	4.3	1.39	pos	9	2	0	1	0	0	0
9		66/F	165	29	ND	0.4	neg	27	0	11	4	0	2	55
10		66/M	311	19	ND	0.6	pos	17	0	7	0	0	0	20
11		70/M	202	28	12	2.09	pos	12	1	1	0	0	3	0
12		68/M	158	40	ND	1.8	pos	24	0	10	0	0	3	30
13	IgA	45/F	88	67	ND	7	neg	28	1	19	1	0	4	40
14		84/F	310	12	11	4.93	pos	7	0	2	0	0	1	10
15		56/M	94	> 60	3.3	0.3	pos	15	0	8	0	0	0	20
16		39/F	135	43	11	1.78	pos	21	8	5	0	0	0	50
17		43/F	56	> 60	4.8	0.6	pos	17	3	3	0	0	0	30
18		74/M	180	34	15	0.7	pos	14	1	0	0	0	1	50
19		19/F	62	> 60	4.5	3	pos	44	0	12	0	0	0	15
20		19/F	57	> 60	3	1.0	pos	34	0	2	0	0	0	10
21		23/M	91	> 60	5.9	0.5	pos	52	0	7	0	0	0	0
22		64/M	99	> 60	5.1	0.4	pos	8	0	4	0	0	0	45
23		72/F	250	17	ND	2	pos	12	0	4	0	0	1	30
24		64/F	108	47	12	1.88	pos	9	0	0	1	0	1	0
25	Lupus	37/F	159	36	8.5	8.0	pos	27	0	16	3	0	3	0
26		40/F	107	56	10.2	4.05	pos	19	0	6	0	0	4	30
27		44/M	180	38	ND	4.6	pos	14	0	3	0	0	4	40
28		51/M	97	> 60	7.6	5.0	pos	47	1	9	0	0	2	10
29		52/M	102	> 60	6.88	5.0	pos	45	2	20	0	0	6	20

To define more precisely the profile of DDR1 expression during the sequence of morphological changes observed in crescent formation, we performed IHC on lupus nephritis class IV-G (A/C) biopsies. Lupus nephritis biopsy samples can display glomerular lesions that are morphologically heterogeneous, consistent with various stages of crescentic lesions. Furthermore, crescent formation results from the serial participation of several different cell types, including macrophages, glomerular parietal epithelial cells, glomerular visceral epithelial cells (podocytes), renal progenitor cells and interstitial fibroblasts [15]. With this in mind, serial sections of lupus nephritis biopsies were immunostained with DDR1 and four different cellular markers: the monocyte/macrophage marker CD68, the podocyte marker nestin, and the PEC markers cytokeratin (CK) 8–18 and CK 19 (Fig. 2B, C). In the very initial stage of cellular crescent formation, characterized by fibrin deposition within the glomerular capillary lumen and only 2–3 layers of cells within the Bowman’s space, DDR1 staining was detected in numerous cells of the glomerulus. These cells were most likely injured podocytes, indicated by similar nestin immunostaining and by abnormal morphologic aspects (Fig. 2B, C line a). It should be noted that the DDR1^+^/Nestin^+^ cells, localized anterior to the emerging cellullar crescent, were also slightly positive for the PEC markers CK 8–18 and CK 19 (Fig. 2C line a). At ther very initial stage of the cellular crescent formation, characterized by gaps in the glomerular capillary wall and plasma proteins and cells within Bowman’s space, DDR1 staining was also detected in injured podocytes, but with a weaker staining intensity (Fig. 2B, C line b). It should be noted that the strongest DDR1 signal was observed in podocytes localized in front of the gap in the glomerular capillary wall, near the Bowman’s capsule or near the growing cellular crescent. Some of these DDR1^+^/Nestin^+^ cells, also positive for CK 8–18, seemed to adhere to both the glomerular basement membrane and the PECs, forming podocyte bridges between the glomerular tuft and Bowman’s capsule (Fig. 2C line b). In a well formed cellular crescent characterized by multiple layers of cells within the Bowman’s space, DDR1 expression was readily detected within a subset of cells of the crescent, mostly located in the periphery of the glomerular tuft (Fig. 2B, C, line c). These DDR1^+^ cells positively immunostained with both PEC markers CK 8/18 and CK 19, but were nestin negative. In a more advanced stage, characterized by fibro-cellular crescent, Bowman’s capsule rupture and periglomerular inflammation, DDR1 immunostaining was detected in the cellular compartment of the crescent but not in the fibrous area (Fig. 2B, C, line d). A similar staining was observed with CK 8/18 and CK 19. Finally, it should be noted that in severe necrotic lesions as observed in patient #6 and #8 with ANCA-associated GN (Table 1), no DDR1 staining could be detected (Additional file 1: Figure S3). This result is not surprising given the degree of structural damage in these glomeruli.

Taken together, our data suggest that DDR1 is de novo expressed during crescent formation, predominantly in injured podocytes, in bridging cells expressing both podocyte and PEC markers, and in a subset of PECs forming the cellular crescent.

### DDR1 expression increases in experimentally-induced crescentic glomerulonephritis and its pharmacological prophylactic inhibition confers morphological and functional protection

**Fig. 3:**
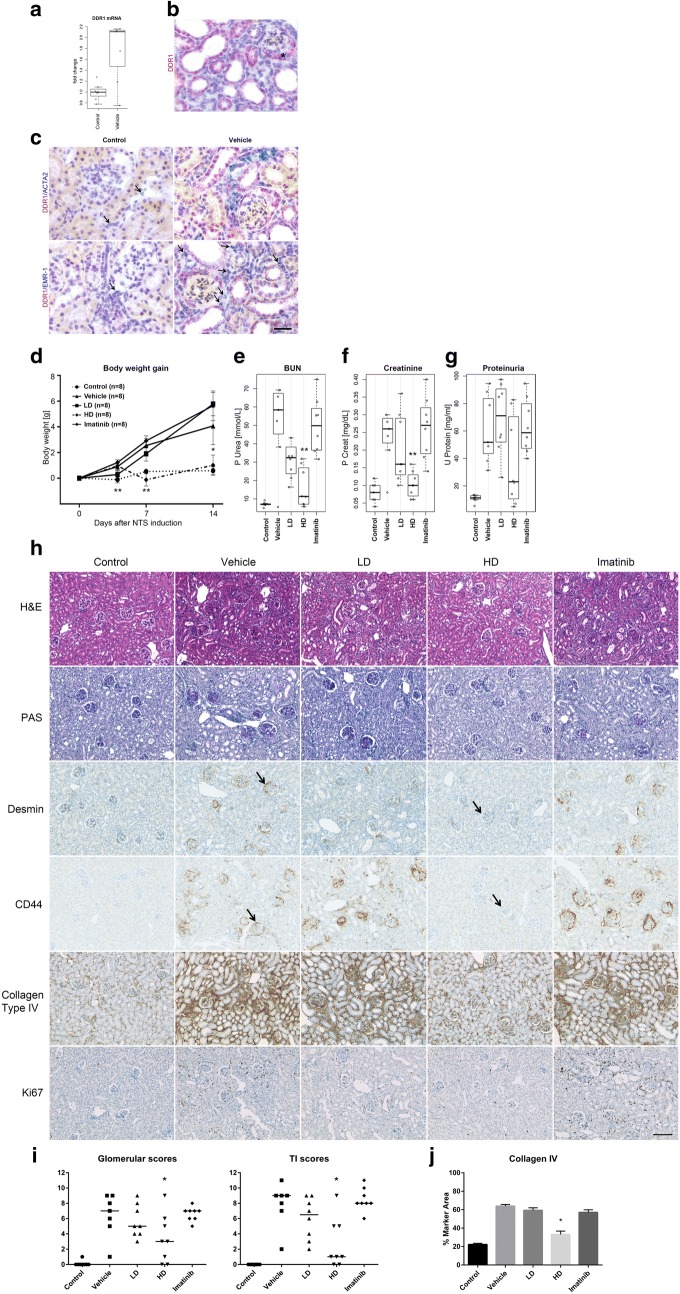
Pharmacological inhibition of DDR1 activation protects animals against NTS-induced crescentic glomerulonephritis. **a** Quantitative RT-PCR for Ddr1 mRNA on whole kidney lysate of control mice (Control) and mice injected with nephrotoxic serum and treated with vehicle (vehicle). **b** Representative Ddr1 in situ hybridization (ISH) performed on tissue harvested from mice 14 days after NTS injection. * = crescent **c** representative DDR1 ISH double labelling with alpha smooth muscle actin (Acta2) or EGF-like module-containing mucin-like hormone receptor-like 1 (Emr-1) in control mice (Control) and mice injected with nephrotoxic serum and treated with vehicle (Vehicle). Arrows = cells labeled with Acta2 or Emr-1. **d**–**g** Body weight evolution (day 1, 4, 7 and 14) and renal function parameters (**e**–**g** blood urea nitrogen (BUN), serum creatinine and proteinuria) measured at sacrifice (day 14). **h** Representative histopathology with Hematoxylin and Eosin (H&E) and Periodic Acid Schiff staining (PAS) and immunohistochemistry for desmin, CD44, Collagen type IV and Ki67. **i** Glomerular or tubulo-interstitial (TI) summary scores from semiquantitative histopathologic evaluation on H&E and PAS stained kidney sections respectively. **j** Morphometry analysis of collagen IV IHC. Statistically significant p value: p < 0.05 = *; p < 0.005 = **. Magnification ×200, scale bar 100 μm

First, we confirmed that DDR1 expression is induced following NTS administration (Fig. 3a–c). Consistently, ISH showed strong DDR1 upregulation in crescents, PECs, and in the tubular structures, most of them displaying lesions characterized by flattening of the epithelium and dilation of the lumen (Fig. 3b). No DDR1 staining was detected in cells co-labeled with Acta2 (myofibroblasts) or Emr-1 (macrophages) (Fig. 3c), indicating that similarly to human GN, DDR1 appears to be predominantly expressed in epithelial cells in the NTS mouse model.

To address the functional significance of DDR1 in experimental crescentic GN, mice were pre-treated with two doses of a very selective DDR1 inhibitor (Roche-Chugai DDR1i), followed by NTS administration. To unravel the specificity of DDR1 activation blockade, a second group was treated with the kinase inhibitor imatinib, which was previously shown to elicit a marked renoprotective effect in the NTS-induced model in Wistar-Kyoto rats [16]. All functional parameters were significantly improved with the high dose of DDR1 inhibitor. The low dose was less efficient, whereas Imatinib failed to show a significant protection (Fig. 3d–g). Histological analyses of kidney sections followed by semiquantitative histopathologic evaluation with glomerular and tubulo-interstitial (TI) scores revealed that functional kidney protection in the high dose DDR1i group was paralleled by tissue preservation of both glomerular and tubulo-interstitial scores (p = 0.02 and p = 0.01 respectively, Fig. 3h–i). In contrast, none of these findings were observed in the Imatinib-treated group (glomerular and tubulo-interstitial scores: p = 0.92 and p = 0.93 respectively). Concordant with glomerular histology, IHC staining with the PEC markers desmin and CD44 revealed marked reductions in the DDR1i HD group (Fig. 3h). Likewise, collagen type IV staining showed a marked reduction of immunoreactivity in the interstitial areas in the DDR1i HD group, but not in the DDR1i LD and Imatinib groups, which were both comparable to the vehicle group. A similar trend was observed in the case of collagen type I (data not shown). Morphometric analysis of collagen type IV staining showed a marked and statistically significant reduction of immunoreactivity in the DDR1i HD group (33.1% ± 3.7 versus 63.9% ± 1.7 for vehicle-treated group, p < 0.001) (Fig. 3j). No significant reduction in collagen type IV staining was observed in the other treated groups. Finally, in agreement with the histology data, quantitation of the cellular proliferation marker Ki67 showed a marked reduction of PEC and tubulo-interstitial staining in the DDR1i HD and DDR1i LD groups (Fig. 3h).

### Selective DDR1 inhibition protects when tested using a therapeutic intervention regime

**Fig. 4:**
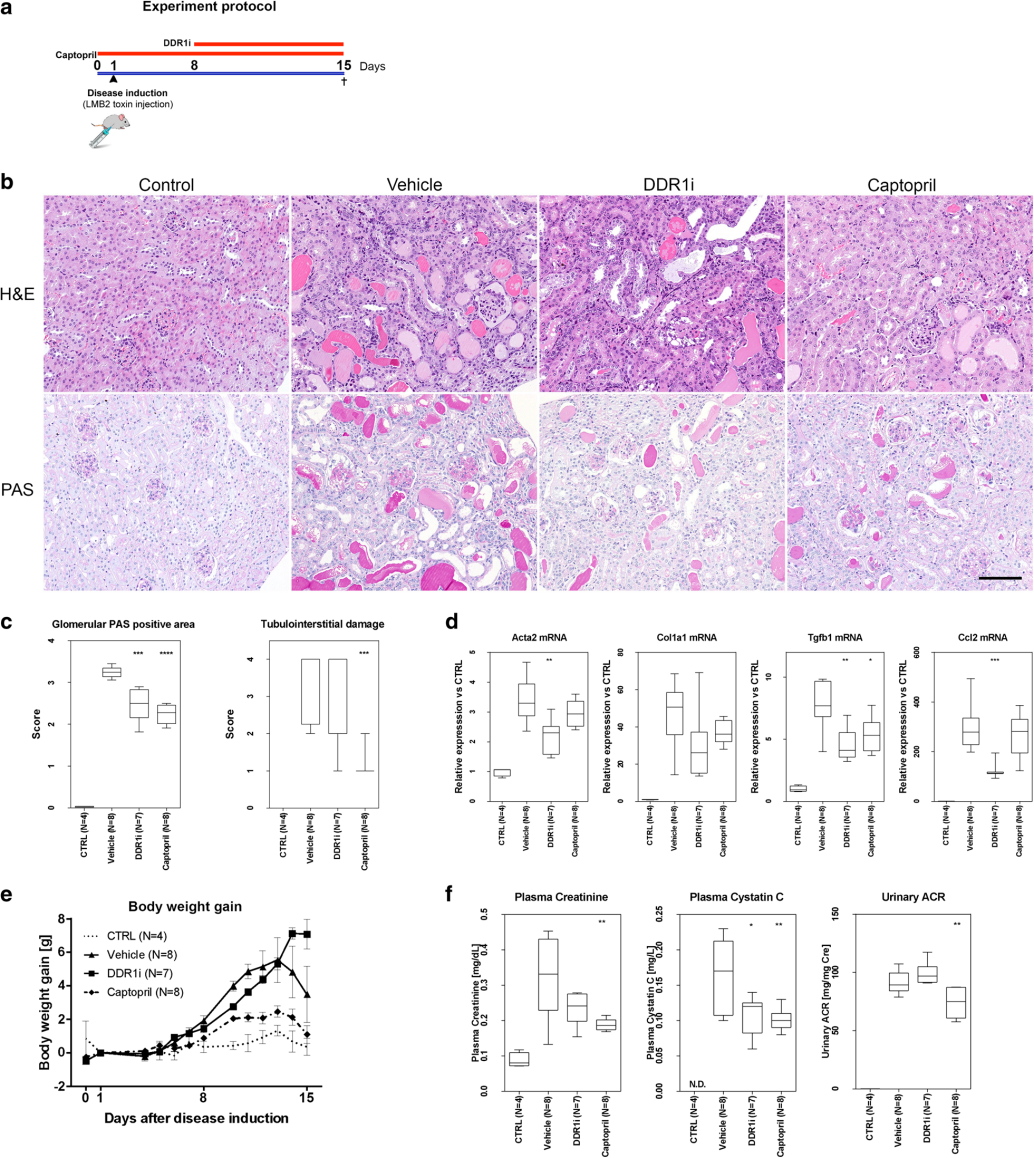
Treatment with DDR1 inhibitor in the NEP25 mouse model of glomerulosclerosis. **a** Schema of the experiment **b** Representative histopathology with Hematoxylin and Eosin (H&E) and Periodic Acid Schiff staining (PAS) in control, vehicle-, DDR1i- and Captopril treated groups at day 15. Magnification ×200, scale bar 100 μm. **c** Semi-quantificative analysis of glomerulosclerosis (glomerular PAS positive area) and tubulointerstitial lesions (tubulointerstitial damage) in control, vehicle-, DDR1i- and Captopril treated groups at day 15. ***p < 0.001, ****p < 0.0001; t-test and Mann–Whitney U test were used for the score of glomerular PAS positive area and tubulointerstitial damage respectively. **d** Quantitative RT-PCR for the fibrosis markers alpha smooth muscle actin (Acta2 mRNA), collagen type 1 (Col1a mRNA) and TGF-β1 (TGF-β1 mRNA), and for the inflammation marker Ccl2 in NEP25 mice treated with DDR1 inhibitor (DDR1i), Captopril or vehicle and in control mice. *p < 0.05, **p < 0.01, ***p < 0.001; t-test. **e** Body weight evolution. **f** Renal function parameters (plasma creatinine and plasma Cystatin C measured at sacrifice) and urinary ACR (24-h urine collection from day 14 to 15 divided by creatinine concentration) in NEP25 mice treated with DDR1 inhibitor (DDR1i), Captopril or vehicle and in control mice (CTRL)

We then aimed to assess the effect of DDR1 inhibition in the context of progressive glomerulosclerosis in the NEP25 mouse model [14]. Mice were treated with the Roche-Chugai DDR1i, Captopril or vehicle (Fig. 4a). Histological and semi-quantitative analyses of glomerulosclerosis in PAS sections and tubulo-interstitial fibrosis/inflammation in H&E sections of kidney tissue at day 15 showed a significant reduction of glomerular PAS positive area in both DDR1i and Captopril groups (p < 0.001 and p < 0.0001 respectively) (Fig. 4B, C). A slight, but not significant, reduction in tubulointerstitial damage was observed in the DDR1i-treated group, whereas this reduction was significant in the Captopril group (p < 0.001) (Fig. 4c). Analyses of several fibrosis and inflammation markers by qRT-PCR in renal cortical tissues at day 15 showed that DDR1i induced a marked reduction of alpha smooth muscle actin (p < 0.01), collagen type 1, TGF-β1 (p < 0.01) and of Ccl2 (p < 0.001) in a range similar to that observed in Captopril-treated mice (Fig. 4d). Captopril was more efficient in improving renal function (Fig. 4e, f)due to the fact that Captopril administration started before disease initiation (preventive approach), whilst the DDR1 inhibitor was only administered during the progression of nephropathy (curative or interventional approach).

### DDR1 inhibition modulates specific gene networks in experimentally-induced crescentic glomerulonephritis

**Fig. 5:**
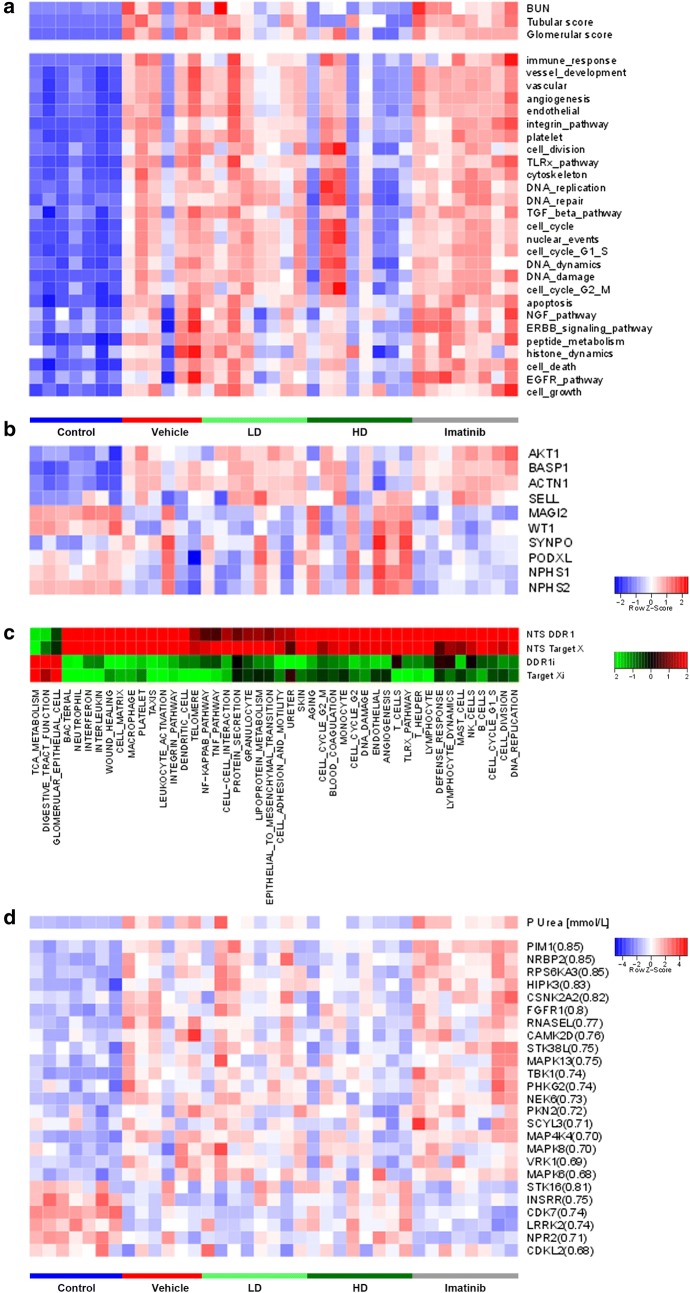
Gene expression profile in NTS-treated mice. **a** Unsupervised gene signature analysis and GSEA using Gene Ontology processes as input genesets. **b** Glomerular cell signature with individual component genes. **c** Comparison of different target modulation in NTS. Heatmap showing differential modulation (by DDR1i or Target X) of the gene networks modulated by NTS induction compared to control. **d** Heatmap showing kinome expression modulation in the different treatment conditions

In order to unveil the mode of action (MoA) of DDR1i, mouse kidneys from the NTS experiment were subjected to gene expression profiling (GEP, GEO Accession Number GSE104426). Unsupervised gene signature analysis and *Gene Set Enrichment Analysis* (GSEA) using Gene Ontology processes as input gene sets showed networks of genes related to immune response, integrin pathway activation and fibrotic processes (TGFβ pathway), all increased in the NTS group (Fig. 5a). In contrast, tissues from mice treated with DDR1i HD showed a significant reduction in the expression of this set of genes (Fig. 5a). This effect was also observed, albeit to a lesser extent, in the DDR1i LD group, but was not in the Imatinib group. Noteworthy, among the weakly perturbed gene signatures, GSEA analyses revealed that the glomerular gene signature, driven by synaptopodin, podocalyxin, NPHS1 and NPHS2 genes, was clearly preserved in the DDR1i HD group, whereas no protection was observed in the case of the Imatinib treated group (Fig. 5b).

Comparison of DDR1i GSEA results with a previous datasets generated in the NTS mouse model treated with other target inhibitors revealed a consistent pattern of biological processes associated with NTS injection that were selectively reverted by DDR1i (Fig. 5c). These include reactivation of genes involved in glomerular epithelial cell processes and deactivation of genes related to wound healing, cell matrix, cell adhesion, and motility and inflammation processes. Thus, to identify the main driver genes specifically modulated by DDR1i treatment, we conducted additional gene level analysis. These data revealed that among the statistically significantly changed genes (n = 785), 175 were identified with a large effect size (logFC ≥ 2) and reverted in the DDR1i group as compared to the NTS group (Additional file 2: Table S3). The highest fraction of perturbed genes (n = 151) was represented by genes exhibiting increased expression upon NTS injection. Genes regulated by treatment with DDR1i were hallmarks in the induction of inflammatory processes, and regulation of cell matrix and adhesion molecules, cell cycle, and apoptosis.

An additional classification criteria (detectable expression in the human kidney epithelial cell line HKC8 [17] or PEC [18] was adopted to further narrow down the gene list (n = 45) to those mechanistically-related to DDR1.

To remove model-specific gene perturbations and identify DDR1 MoA, the differentially expressed genes obtained in the NTS GEP analysis were compared to the genes significantly perturbed in the NEP25 mouse model that were reverted by DDR1i (n = 85). The final intersection revealed 30 genes in common to both models (Additional file 2: Table S4).

Finally, the unexpected lack of therapeutic effect of Imatinib in the NTS model prompted us to conduct a specific kinome analysis to identify potential differences in kinase modulation between DDR1i and Imatinib groups, which could expain the differential therapeutic response. Best BUN-correlated kinases (with a correlation coefficient better than 0.7) were retained and plotted (Fig. 5d). These analyses revealed that 6 kinases were specifically upregulated by DDR1i HD treatment in comparison to Imatinib. In contrast, 19 kinases were downregulated.

Taken together, our data demonstrated that treatment with Roche-Chugai DDR1i reverts inflammatory and fibrotic genes and positively affects genes of the glomerular cell signature particularly podocytes and with an evident effect on the expression of specific kinases.

### DDR1 inhibition decreases PEC pro-inflammatory phenotype in vitro

We then aimed to asses to characterize the impact of DDR1 inhibition on PEC phenotype using a murine PEC cell line [18]. Pre-treatment of PECs for 1 h with DDR1i followed by stimulation with collagen type I for 24 h blocked DDR1 phosphorylation in a dose dependent manner (Additional file 1: Figure S6). Treatment with type I collagen alone induced a significant increase of the matrix metalloproteinases Mmp14 and Mmp2, complement component C3 and vascular cell adhesion molecule 1 (VCAM1) mRNA (Additional file 1: Figure S6B-E). Pre-treatment of the cells with DDR1i caused a dose-dependent inhibition of expression of these genes.

## Discussion

The present study details DDR1 expression in human GN biopsies and demonstrates, for the first time using pharmacological intervention, that selective DDR1 inhibition translates into renal protection. These data confirm the central role of DDR1 in glomerular diseases, previously indicated using gene deletion [8, 12, 13, 19] or gene silencing paradigms [10].

Analysis of human renal biopsies with different types of crescentic GNs shows DDR1 to be strongly expressed in injured podocytes and in PECs forming cellular crescents. Previously, DDR1 has been localized in rat kidney [20] and our data extend that knowledge to humans providing further confidence for the target, indicating that DDR1 expression is restricted to the epithelial compartment (with no DDR1 expression in vessels as reported with the use of unselective antibodies [12]) and providing, for the first time, a detailed analysis of the role of DDR1 in the sequential morphological and cellular events leading to crescent formation in humans. DDR1 was expressed (1) in most injured podocytes during the very initial stage of the disease characterized by glomerular intracapillary immunoinflammatory processes, (2) in bridging cells co-expressing podocyte and PEC markers located in front of, or adjacent to, disruption of the integrity of the glomerular capillary wall, and (3) in a subset of activated/injured PECs forming the cellular crescent, most of these cells being located near the glomerular tuft. Based on this expression analysis, it could be speculated that DDR1 expressed during the initial stage of the crescentic disease in podocytes and bridging cells is reparative but then, when expressed during the active and severe stage of the disease in proliferative PECs is deleterious, participating to excessive *maladaptive* repair processes.

These findings, in addition to previous preclinical evidence of the pathogenic role of DDR1 in crescentic GN [6, 8–10, 12, 13, 21] induced our research group to develop a highly selective DDR1i. Selectivity of DDR1 over the close analogue protein DDR2 is of utmost importance to harness the therapeutic potential of DDR1 antagonism. DDR2 inhibition has in fact been associated with enhanced liver fibrosis (evidence generated by the renowned group of prof. Scott Friedman [6]) and DDR2 activation has been shown to inhibit the development of experimental choroidal neovascularization and retinal neovascularization in mice [7]; thus, inhibition of DDR2 could translate into undesirable side-effects in patients. None of these undesirable side effects were noted in animals treated with the selective DDR1i.

Treatment with our selective DDR1i resulted in preserved renal function and structure, as suggested by histology, IHC and morphometry analyses. Glomerular lesions were reduced, in particular the number of activated PECs as shown by reduced Desmin and CD44 [22] positivity, both recognized markers of an activated PEC phenotype [22, 23]. Tubulo-interstitial inflammation and fibrosis were decreased and the architecture of renal epithelium was maintained. The use of a second model, the NEP25 mouse model [14], was prompted by our interest to further assess the role of DDR1 in a injury model where PEC-induced glomerulosclerosis is caused by podocyte depletion in absence of intra-glomerular inflammation, primary mesangiolysis and primary hypertension thus further strengthening the role of DDR1i in the protection of glomerular function also in a model of focal segmental glomerulosclerosis. Though not as impressive as in the case of NTS, where DDR1i was administrated in a prevention mode, these findings suggest that selective DDR1 inhibition is protective when used in the context of an established lesion (therapeutic regime).

The choice of two different mouse models to investigate the relevance of DDR1 in GNs was consciously made to avoid a model-*biased* interpretation of the results. Since the therapeutic regime was already probed, by one of the co-authors, in the NTS model [19] we chose to generate data in the alternative model of NEP25 [10] to test if treatment could transfer to protection in a different experimental setup. The NEP25 mouse model proved to be extremely robust and was characterized by uniform glomerular injury and sclerosis induced by a simple procedure, i.e., a single intravenous injection of LMB2 toxin. Indeed the model perfectly fits a drug discovery program.

Genomic analysis of the NTS experiment, performed in the context of a drug discovery program, aiming to identify biomarkers exploitable in clinical studies allowed us to suggest that the observed functional and organ protection is due to reduced inflammation and fibrosis.

To specifically decode DDR1 molecular MoA, we took advantage of data generated in the NTS mouse model with compounds modulating other pathways (not DDR1-dependent) and used them to filter and identify a consistent pattern of biological processes associated with renal injury and specifically reverted by DDR1 antagonism. Gene and pathway level analysis identified DDR1 driver genes associated with cell cycle and cell matrix and adhesion, some amongst them were expected for example different collagen genes (*Col1A1, Col1a2, Col3a1, Col6a2*), or other extracellular matrix related proteins such as TIMP1 and FN1, but also a marked reduction of LTBP2 suggestive of a reduced state of activation of epithelial cells. Interestingly, *Cd44*, a hallmark of activated PECs was observed among the top DDR1i modulated genes. Surprisingly, unsupervised gene signature analysis identified podocyte-specific genes as being clearly protected by DDR1i treatment. This is quite remarkable since GEP were generated using whole kidney lysates. These molecular findings are concordant with the IHC results demonstrating PEC de-activation. To support that notion, we also performed a series of experiments showing that DDR1 inhibition blocks activation of PEC in vitro. Taken together, the in vivo and the in vitro evidence seem to suggested that PEC de-activation could be a key part of DDR1i MoA.

One of the major strengths of the present work is that for the first time pharmacological intervention targeting predominantly the renal epithelium can reduce both inflammation and fibrosis. These effects seem to occur without directly affecting fibroblast to myofibroblast activation or the inflammatory component as DDR1 expression was not observed in the relevant cell types. We are however conscious that the lack of immunohistochemical staining does not exclude these additional potential effector cells, highly relevant to the two models studied. Our hypothesis is concordant with a recently published study demonstrating that selective activation of EGFR in renal epithelial cells (proximal tubules) is sufficient to induce tubulo-interstitial fibrosis [24]. As shown by our molecular genomics analysis of NTS kidney samples, DDR1 antagonism has a direct effect in the preservation of the podocyte network and in the de-activation of PECs. It is possible that DDR1 antagonism could reduce an excessive “response to injury” occurring within the glomerular compartment in case of crescentic lesions, and having deleterious consequences on tubulo-interstitial compartment. Those speculations will have to be further addressed by deeper mechanistic studies critical to comprehend the role of DDR1 in glomerular repair and regeneration processes.

Moreover, we can’t ignore that our IHC data seem to suggest a broader role for DDR1 in kidney. We have in fact observed a clear DDR1 tubular upregulation in human crescentic GN as well as in mouse models of glomerular disease. It is interesting to note that DDR1 genetic ablation in other models of chronic, acute or genetic renal disease, such as hypertensive nephropathy, Alport’s syndrome and unilateral ureteral obstruction, has been shown to be protective [3, 19]. Causal association between epithelial cell injury and renal function deterioration, paralleled by fibrosis might be an integral part of the DDR1 MoA in GN. Certainly, this hypothesis deserves further investigation using dedicated mouse models and a detailed IHC characterisation in particular in the context of tubulopathies, acute renal ischemic lesion or transplant reperfusion and/or in general in other ischemic conditions.

It should also be mentioned that when designing the preclinical experiments we sought to add control compounds to validate the models adopted. For that reason, the control compounds (Imatinib and Captopril, respectively used in the NTS and NEP25 models) were administered in a prevention mode to insure better protective performance and consequently better model validation. The doses of both compounds were selected using data reported in the literature for the selected models. Lack of efficacy of Imatinib was surprising since efficacy has been reported at the selected dose [25]. Theoretically this dose has in vivo exposure similar to the DDR1 HD group based on reported in vitro DDR1 potency [26] and in vivo pharmacokinetic data (due the better bioavailability and reduced clearance of Imatinib compared to the DDR1 HD). The lack of efficacy of Imatinib, might suggest that selective inhibition of DDR1 and not of a broader panel of kinases might be a crucial element to achieve kidney protection. Comparative kinome analysis between DDR1i and Imatinib extracted from our GEP indicates that lack of efficacy of Imatinib might possibly due to antagonism of kinases whose activation is key to confer protection to injury, such as STK16, INSSR or CDK7. This hypothesis might also suggest an interesting role for those kinases in GN.

## Conclusion

In conclusion, in the present work, pharmacological inhibition of DDR1 phosphorylation emerges as a unique and differentiated pharmacological therapeutic intervention capable of preserving renal function in both prevention and therapeutic regimens, acting on the renal epithelium and resulting in reduced inflammation and fibrosis in the surrounding interstitial space. Such an intervention paradigm, when available in the clinics, would provide a novel pharmacological tool for combination therapy with anti-inflammatory, endothelial protective or myofibroblast blocking agents.

## Methods

### DDR1 isoform RT-PCR

RNA was purified from normal, ADPKD and ESRD tissue sections using a Tissue Lyser protocol (Qiagen) by adding 350 µl ‘RLT buffer’ plus 350 µl 70% ethanol and vortexing. The sample was then transferred to an RNeasy column and centrifuged at 8000×*g* for 15 s at room temperature. RNase free DNase 1 (80 µl) was added to the column and incubated at room temperature for 15 min. The column was then washed by adding 350 µl RLT buffer and centrifuged at 8000×*g* for 15 s at room temperature. This was repeated with ‘RPE buffer’ (500 µl) twice where the second spin was prolonged to 2 min. Following this the RNaeasy column was transferred to a new microtube and centrifuged at 11,000×*g* for 1 min. The RNAeasy tube was once again transferred to a new microtube and the RNA eluted by adding 50 µl H_2_O and centrifugation at 8000×*g* for 1 min. The elution wash was repeated once and the eluate pooled. Absorbance was measured at 230, 260 and 280 nm in a spectrophotometer and quality approved if the ratios 260/280 and 260/230 were both greater than 1.8.

DDR1 splice isoform specific qRT-PCR assays have been developed for DDR1-variant 3 (DDR1_V3), DDR1-variant 4 (DDR1_V4) and DDR1-variant 5 (DDR1_V5), DDR1-variant 6 (DDR1_V6) which specifically detect one variant. Due to close sequence homology PCR assays for DDR1-variant 1/2 (DDR1_V1/2), DDR1-variant 1/6 (DDR1_V1/6) detect both isoform (see Additional file 3: for primer/probe sequences). All PCR assays were developed using synthetic plasmids carrying DDR1 variant specific sequences assuring comparable sensitivity, specificity, PCR amplification efficiency (see Additional file 3: for plasmid sequences).

Reverse Transcription of RNA was accomplished using the SuperScript III First-Strand Synthesis Kit (Invitrogen) according to the manufacturers’ protocol with a total RNA input of 100 ng per reaction. cDNA samples were then diluted 1:3 in TE buffer and 14 cycles of pre-amplification were carried out using 2× TaqMan PreAmp Master Mix (Applied Biosystems) and pooled DDR1 splice variant specific assays at a final concentration of 0.2× per assay. The following thermocycler program was used: 95 °C for 10 min, followed by 14 cycles at 95 °C for 15 s and 60 °C for 4 min. Pre-amplified cDNA products were diluted 1:5 in TE buffer. qPCR was performed using the 96.96 dynamic array (Fluidigm Corporation, CA, USA) following the manufacturer’s protocol (Fluidigm Quick Reference Card, PN 68000130, Rev. B). Briefly, for each sample a 5μl sample mix was prepared with 1× GE Sample Loading Reagent (Fluidigm), 1× Taqman Gene Expression Mastermix (Applied Biosystems) and diluted, pre-amplified cDNA. For the assay mix 1× Assay Loading Reagent (Fluidigm) was mixed with each of the Taqman Assays (final concentration: 10×), respectively. Priming of the Fluidigm array with control line fluid and mixing of sample and assay reagents was done with an IFC controller. qPCR was performed using the BioMark Instrument with the following cycling parameters: 95 °C for 10 min, followed by 40 cycles at 95 °C for 15 s and 60 °C for 1 min. Data was collected and analyzed with the Real-Time PCR Analysis Software (Fluidigm Corporation, CA, USA). Normalization was performed using geometric mean expression of housekeeping genes B2M, GAPDH, GUSB: ∆Cq = Cq gene of interest—Cq geomean Housekeepers. Normalized values were transformed into relative expression levels using (POWER; -Cq) calculation. The sum of all DDR1 splice isoform expression levels was set at 100% and DDR1 splice isoforms values calculated in % accordingly. DDR1 splice isoform variant 1 and variant 2 isoform expression was calculated using by subtracting results of DDR1-variant 6 (DDR1_V6) from DDR1-variant 1/6 (DDR1_V1/6) to obtain variant DDR1-variant 1 (DDR1_V1) results which were then used to calculate DDR1-variant 2 (DDR1_V2) results.

### DDR1 in situ hybridization on mouse and human kidney sections

In situ hybridization (ISH) on 4 μm-thick mouse or human kidney sections was performed using the RNAscope^®^ 2.5 HD Duplex Assay from Advanced Cell Diagnostics (California, USA) and the DDR1 probes according to the manufacturer’s instructions: mouse DDR1 (RNAscope^®^ Probe-Mm-Ddr1-C2) and human DDR1 (RNAscope^®^ Probe- Hs-DDR1-C2, target region 285—2016, #593591-C2). Double ISH was performed on mouse kidney sections using mouse DDR1 probe and mouse Acta2 (RNAscope^®^ Probe- Mm-Acta2-C2) or mouse Emr1 (RNAscope^®^ Probe- Mm-Emr1). Images were acquired with an Olympus VS120 scanner (Olympus AG, Switzerland) equipped with a VC50 camera and 20× objective.

### Generation of a selective anti-DDR1 monoclonal antibody (DDR1 Rab-819) for IHC analysis

New Zealand White (NZW) rabbits were used for immunization with extracellular domain (ECD) DDR1 protein (R&D Bio-Tech, Zug, Switzerland). The immunization protocol included the repeated injection of immunogen emulsified with CFA into the same animal rotating different application routes. 10 ml peripheral whole blood were collected and used for B cell isolation followed by B-cell culture. After total RNA isolation, cDNA was generated by reverse transcription of the mRNA followed by PCR amplification of the V regions of the rabbit B cells using appropriate primers. The DNA sequences encoding the VHs and VLs were obtained by sequencing the PCR products. Prototype cDNA expression plasmids were used for the recombinant expression of the HC and LC of monoclonal rabbit antibodies that are then expressed transiently in HEK293 cells. After 7 days, the culture supernatants were harvested, purified by Protein A column standard protocols and analyzed for antibody content and specificity on human pancreatic cancer CFPAC-1 and PANC-1 cells.

### Anti-DDR1 antibody specificity testing using DDR1 or DDR2 expressing cancer cell lines

Human pancreatic cancer CFPAC-1 and PANC-1 cells were a gift from Dr. Howard Crawford, University of Michigan. CFPAC-1 cells were maintained in IMDM supplemented with 10% fetal bovine serum 1% l-glutamine and 1% streptomycin/penicillin antibiotics, and PANC-1 cells in DMEM High Glucose supplemented with 10% fetal bovine serum 1% l-glutamine and 1% streptomycin/penicillin antibiotics. Culture media and supplements were purchased from Thermo Fisher Scientific (Waltham, MA). Eight-well Permanox Lab-Tek chamber slides were purchased from NUNC (Cat.# 177445). ImmPRESS™ Reagent (Anti-Rabbit Ig) (Cat. # MP-7401) and ImmPACT™ DAB Peroxidase Substrate (Cat. # SK-4105) were purchased from Vector Laboratories (Burlingame, CA). A rabbit monoclonal antibody against DDR1 (D1G6) was purchased from Cell Signaling Technology (Danvers, MA) and a monoclonal antibody against β-actin was purchased from Sigma (St. Louis, MO). Mayer’s hematoxylin (Cat.# HMM999) was purchased from Scytek and Crystal Mount™ Aqueous Mounting Medium (Cat.# C0612) from Sigma (St. Louis, MO). Immunoblot analyses and cell immunostaining were performed to assess antibody specificity and sensibility.

For immunoblot analyses, cells were lysed in RIPA buffer (50 mM Tris–HCl, pH 7.4, 150 mM NaCl, 1% NP-40, 0.25% sodium deoxycholate and 1.0 mM EDTA) supplemented with protease inhibitors on ice for 1 h. The cell lysates were cleared by centrifugation at 14,000*g* at 4 °C for 15 min, and protein concentration was determined using the BCA kit (Pierce). 20–40 µg of protein lysate were mixed with 1× reducing Laemmli SDS-sample buffer. After a brief centrifugation, the supernatants were boiled and resolved by reducing 7.5% SDS-PAGE, followed by immunoblot analyses using anti-DDR1 antibody D1G6 or SC532. The blots were reprobed with antibodies against β-actin for loading control. For positive control of DDR1 immunoreactivity, we used a lysate of human prostate cancer PC3 cells overexpressing DDR1b, which was generated in our laboratory.

For immunostaining, CFPAC-1 or PANC-1 cells (5 × 10^4^/250 µl of complete medium) were seeded in 8-well chamber slides. Twenty-four hours later, the media were removed and the cells were fixed with cold (− 20 °C) methanol. The cells were then washed (3×) with PBS and then incubated with 3% H_2_O_2_ in PBS for 20 min at room temperature (RT) followed by two washes with PBS. The cells were incubated with 2.5% normal horse serum for 20 min at RT. The serum was removed and the cells were incubated with either in house raised DDR1 antibody (1:50) or control rabbit IgG (1:50) diluted in PBS. After an overnight incubation at 4 °C, the cells were washed with PBS and incubated with the ImmPRESS™ Reagent for 30 min at RT. Detection and visualization of antibody binding was assessed using ImmPACT™ DAB Peroxidase chromogenic substrate, according to the manufacturer instructions. The slides were counterstained with Mayer’s hematoxylin for 5 s, followed by differentiation with tap water for 1 min, and finally covered with a thin layer of Crystal Mount™ Aqueous Mounting Medium. Stained cells were photographed using a Zeiss Axioplan 2 microscope (Zeiss, Gottingen, Germany) equipped with a software-controlled digital camera (Axiovision; Zeiss).

### Human renal tissues

Human renal tissue, fixed in formaldehyde and embedded in paraffin, was selected from the files of the Service of Pathology, University Hospital Geneva: five control normal renal tissues were obtained from patients with nephrectomy performed for neoplasia, and 29 biopsy specimens were obtained from patients with crescentic glomerulonephritis (GN): 5 Goodpasture’s syndrome, 7 ANCA-associated GN, 12 IgA GN, and 5 lupus GN. For all biopsy specimens, standard analysis using light microscopy, immunofluorescence (with anti-immunoglobulin Ig A, G, M, and anti-complement C1q, C3, C4c, and C5b-9 antibodies), and electron microscopy were performed. For all biopsy specimens, standard analyses were performed. Each patient gave informed consent before enrollment. The institutional ethical committee board approved the clinical protocol (CEREH Number 03-081). The research was performed according to the Helsinki’s declaration principles.

### Immunohistochemistry on human tissues

Immunohistochemistry was performed as follows: after antigen heat retrieval, 3 μm sections of the formaldehyde-fixed, paraffin-embedded biopsy specimens were incubated with our specifically in-house raised rabbit monoclonal anti-human DDR1 antibody at a 1:100 dilution 1 h at room temperature followed by an anti-rabbit antibody for 30 min (room temperature) and then liquid diaminobenzidine substrate–chromogen system (DakoCytomation, Glostrup, Denmark). For tubular colocalization experiments in the normal kidney, double immunostaining was performed on serial sections (3 μm thick) using DDR1 and each of these 4 different antibodies: mouse monoclonal anti-human Tamm–Horsfall protein (Technically Speaking, Ontario, Canada) at a 1:80 dilution, mouse monoclonal anti-human Calbindin D (clone CB-955, Sigma-Aldrich, St. Louis, MO) at a 1:800 dilution, rabbit polyclonal anti-human Aquaporin 2 (Abcam, Cambridge, UK) at a 1:20 dilution and rabbit polyclonal anti-human Megalin (anti-LRP2, Sigma-Aldrich) at a 1:5000 dilution. Briefly, 3 μm sections of paraffin-embedded kidneys were submitted to the appropriate antigen retrieval and incubated with DDR1 at a 1:100 dilution 1 h at room temperature followed by an anti-rabbit antibody for 30 min (room temperature) and then liquid diaminobenzidine substrate–chromogen system (DakoCytomation, Glostrup, Denmark). Sections were then incubated with the adequate antibody for 1 h at room temperature followed by the appropriate second antibody for 30 min and then by phosphatase alkaline-fast red enzyme system (DakoCytomation, Glostrup, Denmark).

For DDR1 expression experiments during crescent formation, immunostaining was performed on serial sections of 2 lupus nephritis class IV-G (A/C) biopsies (patients #25 and #29) using DDR1 and each of these 4 different antibodies: mouse monoclonal anti-human CD68 (DakoCytomation, Glostrup, Denmark) at a 1:100 dilution, mouse monoclonal anti-human nestin (R&D systems Bio-Techne, Minneapolis. MN) at a 1:750 dilution, mouse monoclonal anti-human cytokeratin 8 and 18 (BioGenex, Fremont, CA) at a 1:20 dilution and mouse monoclonal anti-human cytokeratin 19 (DakoCytomation, Glostrup, Denmark) at a 1:20 dilution. In addition, some serial sections were immunostained with E-cadherin (Novocastra, Newcastle, UK) at a 1:20 dilution. Briefly, 3 μm sections of paraffin-embedded kidneys were submitted to the appropriate antigen retrieval and incubated with each antibody 1 h at room temperature followed by the appropriate secondary antibody for 30 min (room temperature) and then liquid diaminobenzidine substrate–chromogen system (DakoCytomation, Glostrup, Denmark).

Counterstaining was performed using Mayer hematoxylin. For CD68 and E-cadherin, immunostaings, sections were counterstained with Periodic Acid Schiff (PAS) staining. Stained sections were examined with a Zeiss microscope.

### Pharmacokinetic studies

The route of administration and dosing-regimes for in vivo studies study were based on pharmacokinetic studies. Roche-Chugai DDR1i was formulated as a micro-suspension in a vehicle (7.5% gelatin and 0.9% sodium chloride) and administered by oral application at 10 ml/kg. The oral pharmacokinetics of Roche-Chugai DDR1i was investigated in male 129 Sv mice (Additional file 1: Figure S4). The compound was administered via oral gavage, at 200 mg/kg. Plasma samples (0.05 ml) were collected at 1, 3, 6 and 24 h post-dose (n = 2 mice/time point). Concentrations in mouse plasma samples were determined using a high performance liquid chromatography-tandem mass spectrometry (HPLC–MS/MS) method.

### Roche-Chugai DDR1i in vivo pharmacokinetics and estimated pharmacodynamics

Estimation of inhibition of DDR1-phosphorylation was calculated from the observed plasma concentrations in relationship to the in vitro binding IC50 for Roche-Chugai DDR1i against human DDR1, accounting for mouse plasma protein binding of 98.9% (Additional file 1: Figure S4). Thus, inhibition of DDR1-phosporylation of > 90% in average over 24 h in vivo was estimated from the observed plasma concentrations of Roche-Chugai DDR1i on daily treatment of 129 Sv mice with 200 mg/kg po.

### Kinase assays

The KINOME*scan*™ screening platform (DiscoveRx Corporation, San Diego, USA DiscoverX scanMAX^SM^ Kinase Assay Panel) employing an active site-directed competition binding assay was used to quantitatively measure interactions between the 2 test compounds Roche-Chugai DDR1i and Imatinib on 451 kinases and disease relevant mutant variants. In brief, T7 kinase-tagged phage strains were grown in parallel in 24-well blocks or 96-well blocks in an *E. coli* host derived from the BL21 strain. *E. coli* were grown to log-phase and infected with T7 phage from a frozen stock (multiplicity of infection = 0.4) and incubated with shaking at 32 °C until lysis (90–150 min). The lysates were centrifuged (6000×*g*) and filtered (0.2 μm) to remove cell debris. The remaining kinases were produced in HEK-293 cells and subsequently tagged with DNA for qPCR detection. Streptavidin-coated magnetic beads were treated with biotinylated small molecule ligands for 30 min at room temperature to generate affinity resins for kinase assays. The liganded beads were blocked with excess biotin and washed with blocking buffer [SeaBlock (Pierce), 1% BSA, 0.05% Tween 20, 1 mM DTT] to remove unbound ligand and to reduce nonspecific phage binding. Binding reactions were assembled by combining kinases, liganded affinity beads, and the 2 test compounds in 1× binding buffer (20% SeaBlock, 0.17× PBS, 0.05% Tween 20, 6 mM DTT). Test compounds were prepared as 40× stocks in 100% DMSO and directly diluted into the assay. All reactions were performed in polypropylene 384-well plates in a final volume of 0.02 ml. The assay plates were incubated at room temperature with shaking for 1 h and the affinity beads were washed with wash buffer (1× PBS, 0.05% Tween 20). The beads were then re-suspended in elution buffer (1× PBS, 0.05% Tween 20, 0.5 μM non-biotinylated affinity ligand) and incubated at room temperature with shaking for 30 min. The kinase concentration in the eluates was measured by qPCR. The Selectivity Score (S-score) was calculated for both compounds. The compounds were screened at the concentrations requested, and results for primary screen binding interactions were reported as percent competition (% Competition).

### Nephrotoxic mouse model of crescentic glomerulonephritis

#### Preparation of nephrotoxic serum

Decomplementated nephrotoxic serum (NTS) was prepared as previously described [27]. This protocol has been popularized by Salant and Cybulsky according to the seminal work of Morley and Wheeler in mice [28, 29]. Briefly, sheeps were immunized by subcutaneous injection of mouse isolated glomeruli. The total dose of isolated glomeruli (600 μg) was divided to four injections separated by a 1 week interval. 15 days later and under treatment with anti-allergic agent (Phenergan, Wyeth, USA), sheep received another intravenous injection of the same quantity of mouse glomeruli. One week later animals were sacrificed and serum was collected. Serum was then heat-inactivated at 56 °C for 30 min and filtered (pore size = 0.2 μm) and stored in 1 ml aliquots at − 20 °C. Next we passively induced glomerulonephritis by injecting mice intravenously with the serum collected from sheep. We used four different doses (5, 10, 15, 20 μl/g) for the four serum lots in order to validate the serum and choose the appropriate dose. All serum lots were toxic at doses 10, 15 and 20 μl/g and non-nephrotoxic at 5 μl/g dose. All serums were specific against glomerular compartments (data not shown). One lot was found to induce severe proteinuria with typical histological lesions at 10 μl/g; therefore this serum was used in the next protocol.

### Dose selection for the NTS experiment

DDR1i doses selection was designed based on in vitro pharmacology and bioavailability and clearance obtained in the above mentioned pharmacokinetic studies. Two doses were selected a dose covering 10× the in vitro IC50, resulting in the DDR1 HD dose (200 mg/kg) and a second dose around 1× coverage over IC50, resulting in the DDR1 LD dose (75 mg/kg). The Imatinib dose (60 mg/kg) was simply derived from the literature [25] and was added to the study as pure control since the imatinib in vitro potency and in vivo exposure (data not shown) are in the very same order of the DDR1i HD dose.

### Animal treatment and nephrotoxic serum protocol

All mice were kept in well-controlled animal housing facilities and had free access to water and pellet food. Animal procedures and protocols were in accordance with the European Guidelines for the Care and use of Laboratory Animals and have been approved by the Inserm and UPMC ethical committees.

Glomerulonephritis was induced by retro-orbitaly injection of decomplementated nephrotoxic serum (NTS). A total of 40 female mice 129/SV aged 3–6 months and weighting 18–25 g were used (Janvier, Le Genest-St-Isle, France). The total number of mice was divided into five treatment groups as followed: 8 mice were injected with NTS and fed with vehicle, 8 mice were injected with NTS and fed with low dose DDR1i, 8 mice were injected with NTS and fed with high dose DDR1i, 8 mice were injected with NTS and fed with Imatinib and 8 mice were injected with PBS and fed with vehicle. NTS was injected in mice (10 μl/gBW/day) during 3 consecutive days. Treatment was started 1 day prior first injection of NTS or PBS. All treatments were provided by oral gavage. The average food intake was controlled by weighing the food every 3 days. Mice were found to consume about 4 g/day/mouse which was similar to all groups.

### Urine and plasma sample collection and analyses

All mice were acclimated in metabolic cages for 24 h with free access to food and tap water for 24-h urine collection. Proteinuria, expressed as grams of protein per millimole of creatininuria, was assessed at day 14 using the Pyrogallol Red method and utilizing a KONELAB automate (Thermo Scientific, Waltman, MA, USA). Blood samples were collected on the day of sacrifice (day 14) in EDTA tubes. Creatinine and BUN were assessed in blood plasma and measured using an enzymatic spectrophotometric method and were expressed respectively as mg/dl and mmol/l.

### Animal sacrifice and tissue processing

All mice were sacrificed at day 14 post injection. Kidney tissue was processed after normal saline perfusion as followed. Left kidney: one half was fixed in formalin acetic acid (Formol 2%, acetic acid 5%, alcohol 75% and distilled water 18%) for 12 h and then embedded in paraffin, and the other half was frozen (OCT-embedded). Right kidney: one half was fixed in formalin acetic acid for 12 h and then embedded in paraffin. The other half was cut in two; 1/4 snap frozen, and the other 1/4 was kept in RNA later and frozen in liquid nitrogen.

### Histology and semi-quantitative scoring analyses on NTS mouse tissues

From kidney paraffin blocks, sections of 4 μm were prepared and stained with Hematoxylin and Eosin (H&E) or Periodic Acid Schiff (PAS) and examined under a conventional light microscope (Zeiss Axioskop). The following glomerular findings were appreciated and scored: glomerular hypertrophy, mesangial matrix expansion, hypercellularity and hyperplasia of the parietal epithelial cells and crescents; the following tubulo-interstitial findings were appreciated and scored: tubular degeneration/regeneration, tubular casts, interstitial inflammation and interstitial fibrosis. For severity scoring, the following scores were applied for each mouse tissue: 0 = none; 1 = minimal (approximately 1–20% of the kidney affected; 2 = slight (approximately 21–40% affected); 3 = moderate (approximately 41–60% affected); 4 = marked (61–80% affected); 5 = severe (approximately 81–100% affected). Both glomerular and tubulo-interstitial summary scores were built from the individual scores.

### Immunohistochemistry and morphometric analysis on NTS mouse tissues

On consecutive 4 μm thick slides, immunohistochemistry (IHC) was performed on the Ventana Discovery XT^®^ immunostainer with anti-desmin rabbit polyclonal antibody (Spring Bioscience, E2574, dilution 1:100), anti-CD44 rat monoclonal (BD Pharmingen, 550538, dilution 1:50), anti-collagen type IV rabbit polyclonal antibody (Millipore, AB756P, dilution 1:200) and anti-Ki67 rat polyclonal antibody (eBioscience, 14-5698-82, dilution 1:2000), as primary antibodies in a standard protocol. The Biotin-SP-conjugated AffiniPure donkey anti-rabbit IgG (Jackson ImmunoResearch, 711-065-152, dilution 1:100) for desmin and collagen type IV or the Biotin-SP-conjugated AffiniPure donkey anti-rat IgG (Jackson ImmunoResearch, 712-065-153, dilution 1:100) for CD44 and Ki67 were used as secondary antibody in combination with the Ventana DAB Map^®^ (05266360001) detection kit. Slides were counterstained with hematoxylin. Collagen type IV IHC slides were scanned with an Aperio ScanScopeAT^®^ slidescanner. Quantitative morphometry analysis was performed with a rule set recognizing the percentage area of collagen type IV staining using the Definiens TissueStudio^®^ Version 3.51 software. All images were taken at 20×.

### NEP25 mouse model of glomerulosclerosis

NEP25 mice were kindly provided from Prof. Matsusaka in Tokai University. All mice were kept in well-controlled animal housing facilities and had free access to water and pellet food. Animal procedures and protocols were in accordance with the Guidelines for the Care and Use of Laboratory Animals at Chugai Pharmaceutical Co. Ltd. and approved by Institutional Animal Care and Use Committee.

A total of 28 male mice (4 C57BL/6NCrlCrlj mice and 24 Nephrin-hCD25 (NEP25) transgenic mice) aged 10 weeks were used. One day before LMB2 (Anti-Tac (Fv)-PE38) injection, NEP25 mice were divided into three groups of 8 mice each based on bodyweight as followed: one group with DDR1i treatment, one group with Captopril treatment and one group receiving vehicle. 0.7 ng/g BW of LMB2 was intravenously injected in NEP25 mice to induce glomerulosclerosis (day 1). Captopril treatment (0.15 mg/mL in drinking water, approximatively 30 mg/kg/day) was started 1 day before LMB2 injection and given until sacrifice. DDR1i treatment (50 mg/kg/day) and vehicle were started 7 days after LMB2 injection. DDR1i and vehicle were provided by oral gavage. All mice were acclimated in metabolic cages with free access to food and tap water. Body weight was measured daily. A 24-h urine was collected from day 14 to 15. Albumin and creatinine were measured in urine by Lbis^®^ Mouse Urinary Albumin Assay Kit using a TBA-120FR (Toshiba Medical Systems Corporation). Albumin-creatinine ratio (ACR), expressed as mg of albuminuria/mg of creatininuria, was assessed at day 15. All mice were sacrificed at day 15 post injection. It should be mentioned that one mouse in the DDR1i group was excluded from the analysis because congenital renal anomalies were found at autopsy (DDR1i group N = 7). Blood samples were collected and mixed with heparin. Creatinine was measured by HPLC and expressed as mg/dl. Cystatin C was measured using Iatro CysC (LSI Medience Corporation) and expressed as mg/L. Both kidneys were removed and processed as followed: one-quarter of each kidney was fixed in either 10% Formalin Neutral Buffer Solution (for H&E staining) or Methyl Carnoy’s fixative (for PAS staining) and then embedded in paraffin. Cortex of the rest kidney was snap frozen in liquid nitrogen for mRNA analysis. PAS stained sections were analyzed for quantifying glomerular damage. PAS positive area was graded for each glomerulus using a score of 0–4 as previously described [14]. Briefly, more than 50 of randomly selected glomeruli from each mouse were evaluated for glomerulosclerosis. Score 0 represents no lesion, whereas 1, 2, 3, and 4 represent mesangial matrix expansion, hyalinosis, or sclerosis, involving ≤ 25, 25% to ≤ 50, 50% to ≤ 75 and > 75% of the glomerular tuft area, respectively. Tubulointerstitial damage was graded for each mouse on H&E-stained sections, using a score of 0–4. Score 0 represents no lesion, whereas 1, 2, 3, and 4 represents lesions of tubulointerstitial fibrosis/inflammation, involving ≤ 25, 25% to ≤ 50%, 50 to ≤ 75%, and > 75% of the cortex tubulointerstitial area, respectively.

Total RNA was isolated with RNeasy MINI kit (QIAGEN, #74106) from frozen cortical kidney lysate and cDNA was synthesized by reverse transcription. qRT-PCR was performed using fibrosis markers *Tgf*-*b1* (Mm01178820_m1, TaqMan^®^ Gene Expression Assays, Applied Biosystems), *Acta2* (Mm00725412_s1) and *Col1a1* (Mm00801666_g1) and of the inflammation marker *Ccl2* (Mm00441242_m1).

### Microarray analysis of gene expression on NTS and NEP25 mouse tissues

Harvested tissues were lysed with RNeasy lysis buffer immediately after treatment with MM or DM. Lysates were sheared with QIAshredder spin columns and total RNA was extracted using RNeasy^®^ kits as per the manufacturer’s guidelines (QIAGEN GmbH, Hilden, Germany). Starting with 1 μg total RNA per sample, reverse transcription into cDNA and subsequent steps until hybridization onto GeneChip^®^ oligonucleotide microarrays (Human Genome U133 plus 2.0) and scanning were conducted using the manufacturer’s kits and instructions (Affymetrix Inc., Santa Clara, CA). Quantile normalization using the Robust Multi-array Average (RMA) method was applied to the raw individual microarray data set. The dataset was processed using the standard Bioconductor affy package [30]. After RMA normalization, probes representing the same genes were collapsed into a single value and standardized by taking the mean value for each gene across the sample set. For each treatment group (LD DDR1i, HD DDR1i and Imatinib) contrasts were calculated against the Control and the resulting differentially expressed gene list were subject to Gene Set Enrichment Analysis [31].

#### Gene ontology signature enrichment

Expression signals for each samples was tested for enrichment of gene ontology biological process terms [32] using an implementation of the Wilcoxxon’ test called bioQC [33]. The resulting enrichment scores were taken as a measure of the upregulation/downregulation of a specific biological process.

### In vitro PEC experiments

Mouse primary PEC were plated and rested overnight. Cells were incubated in RPMI-1640 containing 1% serum and supplemented with different concentrations of DDR1 inhibitor (0.01, 0.1 and 1 μM) for 1 h followed by additional treatment with type I collagen 100 μg/mL (Nitta Gelatin, Japan). After 6 or 24 h of type I collagen stimulation, cells were lysed for phospho DDR1 ELISA (Cell Signaling Technology) and total RNA isolation. Total RNA was isolated and amplified using an RNeasy Mini kit (Qiagen) and Transcriptor Universal cDNA Master (Roche) according to manufacturer’s instructions. Quantitative RT-PCR was performed on a LightCycler LC480 (Roche) for C3, Mmp2, Mmp14 and Vcam1 using the following primers: C3 (Mm01232779_m1, TaqMan^®^ Gene Expression Assays, Applied Biosystems), Mmp2 (Mm00439498_m1), Mmp14 (Mm00485054_m1), and Vcam1 (Mm01320970_m1). Relative gene expression was calculated with the 2-∆Ct method using GAPDH as an endogenous control.

### Statistics

Values are expressed as mean ± SEM. Box-and-whisker plot represents quartiles. Data were analyzed using one-way analysis of variance followed by protected least significant difference Fisher’s test of the Stat-view software package. Glomerular and tubular summary scores and collagen type IV quantitative morphometry data were analyzed using the Kruskal–Wallis test followed by p value adjusted Dunett’s post hoc test. Data on in vivo NEP study were analysed using t-test with GraphPad Prism except for the score of tubulointerstitial damage which was analysed by Mann–Whitney U test. Data on in vitro PEC study were analysed using Dunnett’s multiple comparison test with GraphPad Prism. Results with p < 0.05 were considered statistically significant.

## Additional files

**Additional file 1.** Additional figures.

**Additional file 2.** Additional tables.

**Additional file 3.** Additional materials.

## Abbreviations

DDR1: Discoidin domain receptor 1
GN: glomerulonephritis
NTS: nephrotoxic serum
PEC: parietal epithelial cell
RTK: receptor tyrosine kinase
KO: knockout
ASO: antisense oligonucleotides
DDR1i: DDR1 inhibitor
IHC: immunohistochemistry
CK: cytokeratin
GEP: gene expression profiling
GSEA: Gene Set Enrichment Analysis
MoA: mode of action
BUN: blood urea nitrogen

## References

[1] Vogel W (1997). The discoidin domain receptor tyrosine kinases are activated by collagen. Mol Cell.

[2] Valiathan RR (2012). Discoidin domain receptor tyrosine kinases: new players in cancer progression. Cancer Metastasis Rev.

[3] Leitinger B (2014). Discoidin domain receptor functions in physiological and pathological conditions. Int Rev Cell Mol Biol.

[4] Borza CM, Pozzi A (2014). Discoidin domain receptors in disease. Matrix Biol.

[5] Yeh YC, Lin HH, Tang MJ (2012). A tale of two collagen receptors, integrin beta1 and discoidin domain receptor 1, in epithelial cell differentiation. Am J Physiol Cell Physiol.

[6] Hahn WH (2010). Linkage and association study of discoidin domain receptor 1 as a novel susceptibility gene for childhood IgA nephropathy. Int J Mol Med.

[7] Kavvadas P, Dussaule JC, Chatziantoniou C (2014). Searching novel diagnostic markers and targets for therapy of CKD. Kidney Int Suppl.

[8] Flamant M (2006). Discoidin domain receptor 1 null mice are protected against hypertension-induced renal disease. JASN.

[9] Guerrot D (2011). Discoidin domain receptor 1 is a major mediator of inflammation and fibrosis in obstructive nephropathy. Am J Pathol.

[10] Kerroch M (2016). Protective effects of genetic inhibition of Discoidin Domain Receptor 1 in experimental renal disease. Sci Rep.

[11] Alfieri C (2015). Discoidin domain receptor-1 and periostin: new players in chronic kidney disease. Nephrol Dial Transplant.

[12] Kerroch M (2012). Genetic inhibition of discoidin domain receptor 1 protects mice against crescentic glomerulonephritis. FASEB J.

[13] Gross O (2010). Loss of collagen-receptor DDR1 delays renal fibrosis in hereditary type IV collagen disease. Matrix Biol.

[14] Matsusaka T (2005). Genetic engineering of glomerular sclerosis in the mouse via control of onset and severity of podocyte-specific injury. JASN.

[15] Jennette JC (2003). Rapidly progressive crescentic glomerulonephritis. Kidney Int.

[16] Iyoda M (2013). Long- and short-term treatment with imatinib attenuates the development of chronic kidney disease in experimental anti-glomerular basement membrane nephritis. Nephrol Dial Transplant.

[17] Moll S (2013). Epithelial cells as active player in fibrosis: findings from an in vitro model. PLoS ONE.

[18] Kabgani N (2012). Primary cultures of glomerular parietal epithelial cells or podocytes with proven origin. PLoS ONE.

[19] Dorison A, Placier S, Dubois Y, Chladichristos C, Rondeau E, Chatziantoniou C, Dussaule JC (2015). Discoidin domain receptor 1 is a key mediator of ischemia-reperfusion induced injury.

[20] Lee R (2004). Localization of discoidin domain receptors in rat kidney. Nephron. Exp Nephrol.

[21] Rubel D (2014). Collagen receptors integrin alpha2beta1 and discoidin domain receptor 1 regulate maturation of the glomerular basement membrane and loss of integrin alpha2beta1 delays kidney fibrosis in COL4A3 knockout mice. Matrix Biol.

[22] Fatima H (2012). Parietal epithelial cell activation marker in early recurrence of FSGS in the transplant. Clin J Am Soc Nephrol.

[23] Stamenkovic I, Skalli O, Gabbiani G (1986). Distribution of intermediate filament proteins in normal and diseased human glomeruli. Am J Pathol.

[24] Overstreet JM (2017). Selective activation of epidermal growth factor receptor in renal proximal tubule induces tubulointerstitial fibrosis. FASEB J.

[25] Iyoda M (2009). Preventive and therapeutic effects of imatinib in Wistar-Kyoto rats with anti-glomerular basement membrane glomerulonephritis. Kidney Int.

[26] Day E (2008). Inhibition of collagen-induced discoidin domain receptor 1 and 2 activation by imatinib, nilotinib and dasatinib. Eur J Pharmacol.

[27] Salant DJ, Cybulsky AV (1988). Experimental glomerulonephritis. Methods Enzymol.

[28] Lloyd CM (1997). RANTES and monocyte chemoattractant protein-1 (MCP-1) play an important role in the inflammatory phase of crescentic nephritis, but only MCP-1 is involved in crescent formation and interstitial fibrosis. J Exp Med.

[29] Morley AR, Wheeler J (1985). Cell proliferation within Bowman’s capsule in mice. J Pathol.

[30] Gautier L (2004). affy—analysis of Affymetrix GeneChip data at the probe level. Bioinformatics.

[31] Subramanian A (2005). Gene set enrichment analysis: a knowledge-based approach for interpreting genome-wide expression profiles. Proc Natl Acad Sci USA.

[32] Osumi-Sutherland D, Ponta E, Courtot M, Parkinson H, Badi L (2015). Cell, chemical and anatomical views of the gene ontology: mapping to a roche controlled vocabulary. CEUR Workshop Proc..

[33] Zhang JD (2017). Detect tissue heterogeneity in gene expression data with BioQC. BMC Genomics.

