# Supplementary material for: Selective pharmacological inhibition of DDR1 prevents experimentally-induced glomerulonephritis in prevention and therapeutic regime
Source: J Transl Med. 2018 Jun 1;16:148. doi: 10.1186/s12967-018-1524-5 (PMC5984769; doi:10.1186/s12967-018-1524-5)

**Additional file 1: Figure S1**


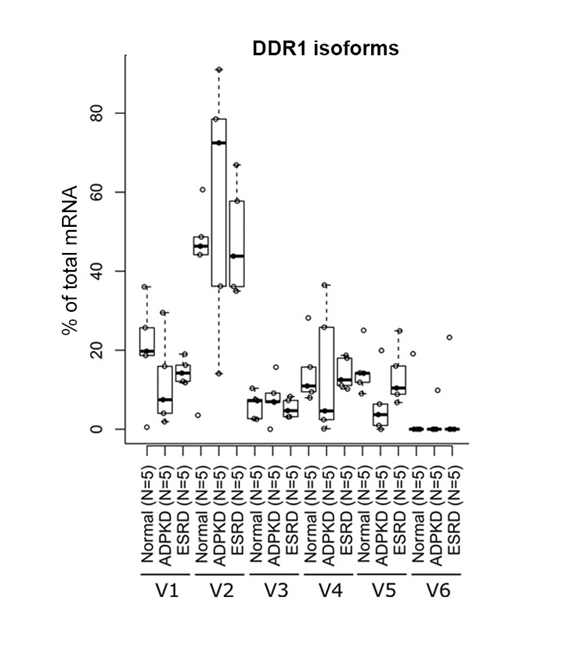


**Additional file 2: Figure S2**


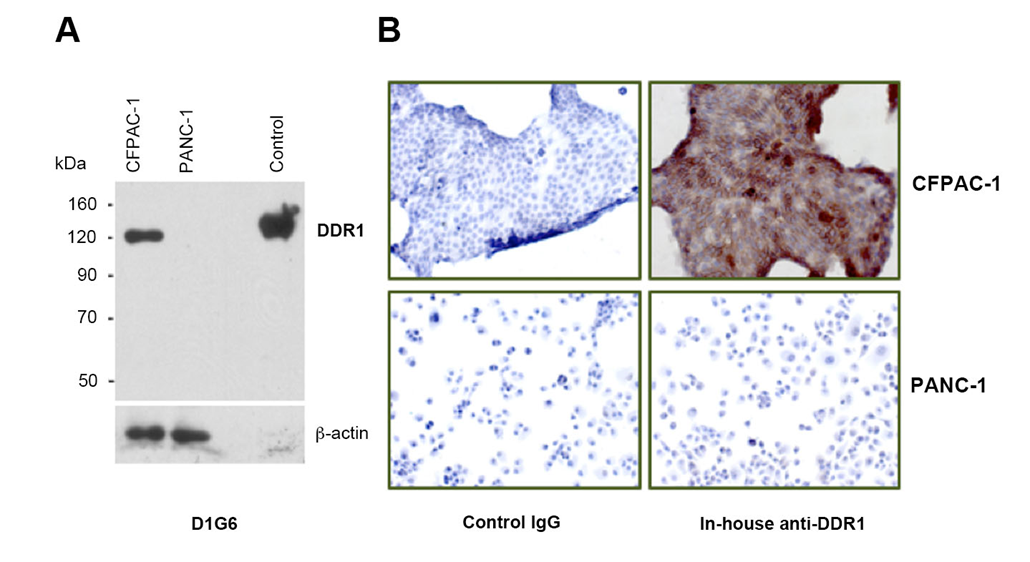


**Additional file 3: Figure S3**


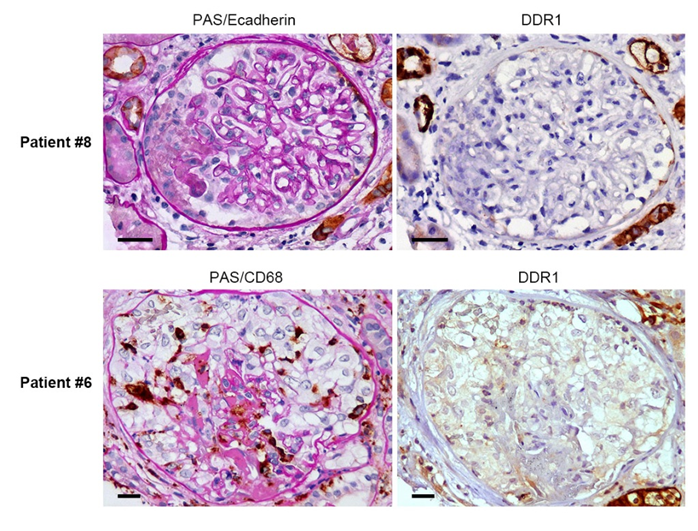


**Additional file 4: Figure S4**


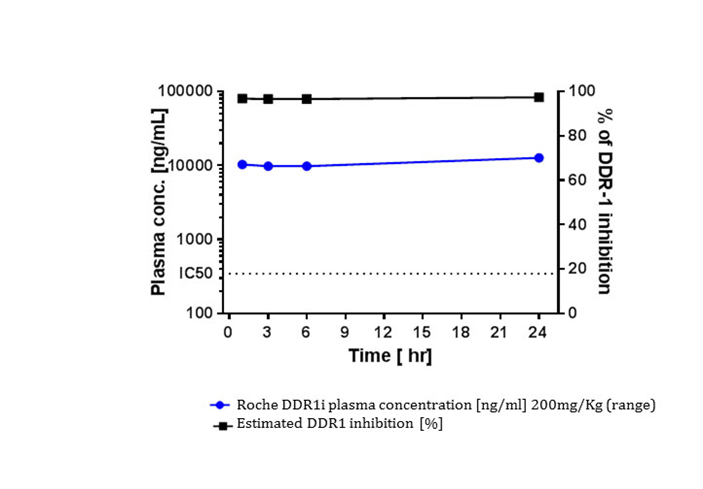


**Additional file 5: Figure S5**


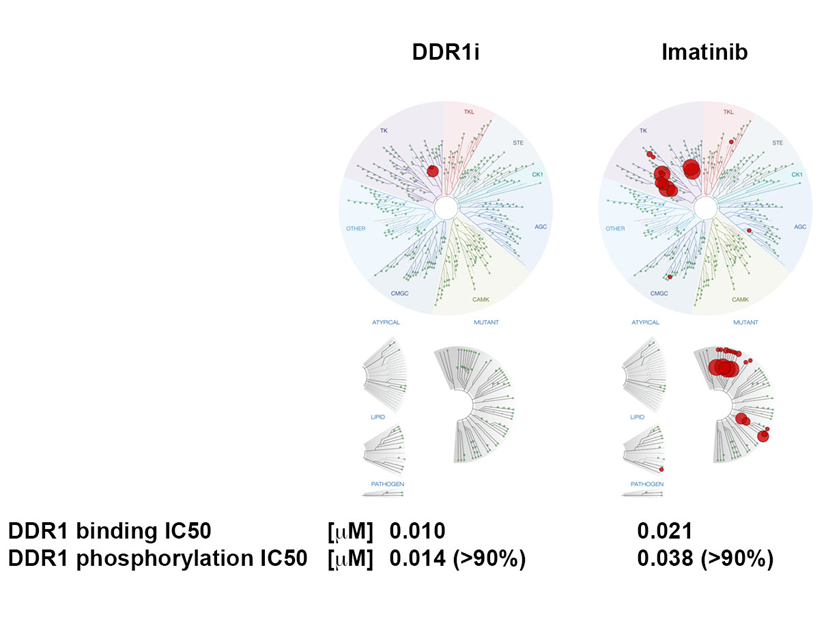


**Additional file 6: Figure S6**


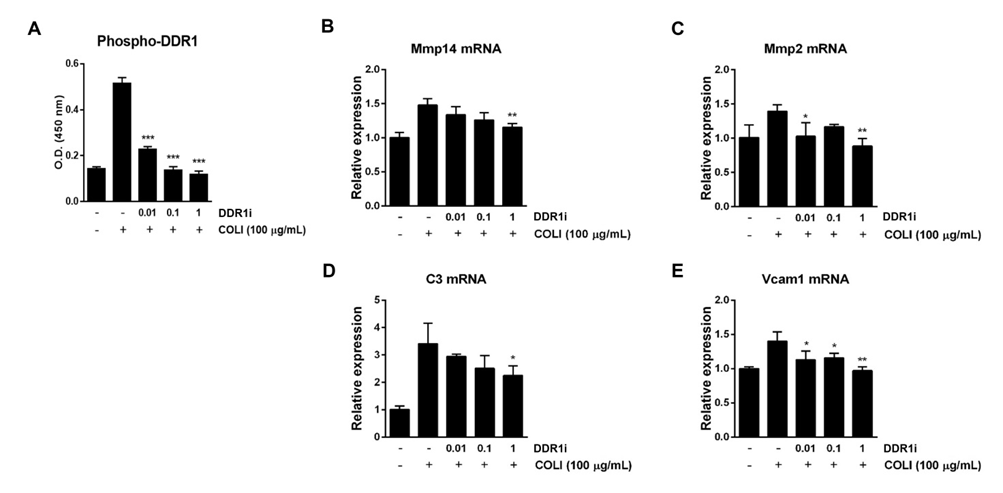

Supplement: Supplementary file 1 — Additional file 1. Additional figures. [file 12967_2018_1524_MOESM1_ESM.docx]
