# Supplementary material for: Selective pharmacological inhibition of DDR1 prevents experimentally-induced glomerulonephritis in prevention and therapeutic regime
Source: J Transl Med. 2018 Jun 1;16:148. doi: 10.1186/s12967-018-1524-5 (PMC5984769; doi:10.1186/s12967-018-1524-5)
Supplement: Supplementary file 2 — Additional file 2. Additional tables. [file 12967_2018_1524_MOESM2_ESM.zip › Supplemental Table 1.docx]

## Additional Table S1

## DDR1 transcript variants and related forward and reverse primers

| **DDR1 Transcript Variant Mix** | **Fwd primer** | **Rev primer** | **Probe** |
| --- | --- | --- | --- |
| 1 and 6 | B1 | A2 | I1 |
| 1 and 2 | E2 | F1 | I4 |
| 3 | C1 | D2 | I2 |
| 4 | B1 | A1 | I1 |
| 5 | B2 | A1 | I1 |
| 6 | E1 | F1 | I3 |

For each primer, numbers are length, Tm and GC%.

NM_013994 and NM_013993 - PRODUCT SIZE: 105 bp

**B1** FORWARD PRIMER 18 60.21 66.67 ACCGCCCAGGTCCTAGAG

**A3** REVERSE PRIMER 18 60.61 61.11 AGAGCAGCAACGCAGAGC

**I1** INTERNAL OLIGO 18 60.42 66.67 CACTCCGCTCCCTGTGTC

NM_001954 and NM_001202523 - PRODUCT SIZE: 107 bp

**B1** FORWARD PRIMER 18 60.21 66.67 ACCGCCCAGGTCCTAGAG

**A2** REVERSE PRIMER 20 62.18 65.00 GTCCCCACTGTAGGCAGAGC

**I1** INTERNAL OLIGO 18 60.42 66.67 CACTCCGCTCCCTGTGTC

NM_001202521 - PRODUCT SIZE: 101 bp

**B1** FORWARD PRIMER 18 60.21 66.67 ACCGCCCAGGTCCTAGAG

**A1** REVERSE PRIMER 18 59.21 55.56 AGGTGCACCAGAGCCATT

**I1** INTERNAL OLIGO 18 60.42 66.67 CACTCCGCTCCCTGTGTC

NM_001202522 - PRODUCT SIZE: 104 bp

**B2** FORWARD PRIMER 21 58.29 57.14 CTCCTCAGCAAGGTCCTAGAG

**A1** REVERSE PRIMER 18 59.21 55.56 AGGTGCACCAGAGCCATT

**I1** INTERNAL OLIGO 18 60.42 66.67 CACTCCGCTCCCTGTGTC

NM_013994 - PRODUCT SIZE: 139 bp

**C1** FORWARD PRIMER 20 61.19 55.00 AGGTCGACAGCCCTCAAGAT

**D2** REVERSE PRIMER 21 59.27 47.62 TCATTCCTGGAGAACAAGGAG

**I2** INTERNAL OLIGO 20 59.95 50.00 CCCTTAATGTGCGTAAGGGA

Forward p. binds to exon 13; Reverse p. binds to exon 14.

NM_013993 and all others except NM_013994 - PRODUCT SIZE: 121 bp

**C1** FORWARD PRIMER 20 61.19 55.00 AGGTCGACAGCCCTCAAGAT

**D1** REVERSE PRIMER 20 60.46 45.00 TCATTCCTGGCATTCTTGGT

**I2** INTERNAL OLIGO 20 59.95 50.00 CCCTTAATGTGCGTAAGGGA

NM_001202523 - PRODUCT SIZE: 135 bp

**E1** FORWARD PRIMER 20 60.70 60.00 CTGCCACTCTAACCCACCAG

**F1** REVERSE PRIMER 20 59.82 60.00 CTCTGGTCCCATAGCTCCTG

**I3** INTERNAL OLIGO 20 60.56 50.00 TGAGAGAATGTCACTGCCGA

Forward p. binds to exon 1, Reverse p. binds to exon 2 of NM_001202523

NM_001954 and NM_013993 - PRODUCT SIZE: 126 bp

**E2** FORWARD PRIMER 20 59.41 55.00 GGAAGAGCGATGAGAGGTGT

**F1** REVERSE PRIMER 20 59.82 60.00 CTCTGGTCCCATAGCTCCTG

**I4** INTERNAL OLIGO 20 59.97 50.00 TGGCTATTCACTGAGCGATG

Forward p. binds to exons 2+3 of NM_001954 and to exons 1+2 of NM_013993, Reverse p. to exon4 of NM_001954 and to exon3 of NM_013993.

Notice that A1, A2, A3, B2, D1 and E2 primers span splice sites.

Primer B1 does not bind to exon 10 of NM_001202522.
