# Supplementary material for: Selective pharmacological inhibition of DDR1 prevents experimentally-induced glomerulonephritis in prevention and therapeutic regime
Source: J Transl Med. 2018 Jun 1;16:148. doi: 10.1186/s12967-018-1524-5 (PMC5984769; doi:10.1186/s12967-018-1524-5)
Supplement: Supplementary file 2 — Additional file 2. Additional tables. [file 12967_2018_1524_MOESM2_ESM.zip › Supplemental Table 2- DDR1 selectivity profile.docx]

**Additional Table S2**

**DiscoveRx selectivity profiles of the *in vivo* PoC experiment compounds (tested at 1 μM)**

|  | **DDR1i** | **Imatinib** |
| --- | --- | --- |
| DiscoveRx Gene Symbol | Percent Competition | Percent Competition |
| AAK1 | <50 | <50 |
| ABL1(E255K)-phosphorylated | <50 | 98 |
| ABL1(F317I)-nonphosphorylated | <50 | 95 |
| ABL1(F317I)-phosphorylated | <50 | 94 |
| ABL1(F317L)-nonphosphorylated | <50 | 75 |
| ABL1(F317L)-phosphorylated | <50 | 96 |
| ABL1(H396P)-nonphosphorylated | <50 | 100 |
| ABL1(H396P)-phosphorylated | <50 | 99 |
| ABL1(M351T)-phosphorylated | <50 | 98 |
| ABL1(Q252H)-nonphosphorylated | <50 | 98 |
| ABL1(Q252H)-phosphorylated | <50 | 99 |
| ABL1(T315I)-nonphosphorylated | <50 | <50 |
| ABL1(T315I)-phosphorylated | <50 | <50 |
| ABL1(Y253F)-phosphorylated | <50 | 98 |
| ABL1-nonphosphorylated | <50 | 100 |
| ABL1-phosphorylated | <50 | 100 |
| ABL2 | <50 | 99 |
| ACVR1 | <50 | <50 |
| ACVR1B | <50 | <50 |
| ACVR2A | <50 | <50 |
| ACVR2B | <50 | <50 |
| ACVRL1 | <50 | <50 |
| ADCK3 | <50 | <50 |
| ADCK4 | <50 | <50 |
| AKT1 | <50 | <50 |
| AKT2 | <50 | <50 |
| AKT3 | <50 | <50 |
| ALK | <50 | <50 |
| ALK(C1156Y) | <50 | <50 |
| ALK(L1196M) | <50 | <50 |
| AMPK-alpha1 | <50 | <50 |
| AMPK-alpha2 | <50 | <50 |
| ANKK1 | <50 | <50 |
| ARK5 | <50 | <50 |
| ASK1 | <50 | <50 |
| ASK2 | <50 | <50 |
| AURKA | <50 | <50 |
| AURKB | <50 | <50 |
| AURKC | <50 | <50 |
| AXL | <50 | <50 |
| BIKE | <50 | <50 |
| BLK | <50 | 99 |
| BMPR1A | <50 | <50 |
| BMPR1B | <50 | <50 |
| BMPR2 | <50 | <50 |
| BMX | <50 | <50 |
| BRAF | <50 | 93 |
| BRAF(V600E) | <50 | 99 |
| BRK | <50 | <50 |
| BRSK1 | <50 | <50 |
| BRSK2 | <50 | <50 |
| BTK | <50 | <50 |
| BUB1 | <50 | <50 |
| CAMK1 | <50 | <50 |
| CAMK1B | <50 | <50 |
| CAMK1D | <50 | <50 |
| CAMK1G | <50 | <50 |
| CAMK2A | <50 | <50 |
| CAMK2B | <50 | <50 |
| CAMK2D | <50 | <50 |
| CAMK2G | <50 | <50 |
| CAMK4 | <50 | <50 |
| CAMKK1 | <50 | <50 |
| CAMKK2 | <50 | <50 |
| CASK | <50 | <50 |
| CDC2L1 | <50 | <50 |
| CDC2L2 | <50 | <50 |
| CDC2L5 | <50 | <50 |
| CDK11 | <50 | 67 |
| CDK2 | <50 | <50 |
| CDK3 | <50 | <50 |
| CDK4 | <50 | <50 |
| CDK4-cyclinD1 | <50 | <50 |
| CDK4-cyclinD3 | <50 | <50 |
| CDK5 | <50 | <50 |
| CDK7 | <50 | <50 |
| CDK8 | <50 | <50 |
| CDK9 | <50 | <50 |
| CDKL1 | <50 | <50 |
| CDKL2 | <50 | <50 |
| CDKL3 | <50 | <50 |
| CDKL5 | <50 | <50 |
| CHEK1 | <50 | <50 |
| CHEK2 | <50 | <50 |
| CIT | <50 | <50 |
| CLK1 | <50 | 76 |
| CLK2 | <50 | <50 |
| CLK3 | <50 | <50 |
| CLK4 | <50 | 88 |
| CSF1R | <50 | 100 |
| CSF1R-autoinhibited | <50 | <50 |
| CSK | <50 | <50 |
| CSNK1A1 | <50 | <50 |
| CSNK1A1L | <50 | <50 |
| CSNK1D | <50 | <50 |
| CSNK1E | <50 | <50 |
| CSNK1G1 | <50 | <50 |
| CSNK1G2 | <50 | <50 |
| CSNK1G3 | <50 | <50 |
| CSNK2A1 | <50 | 95 |
| CSNK2A2 | <50 | 74 |
| CTK | <50 | <50 |
| DAPK1 | <50 | <50 |
| DAPK2 | <50 | <50 |
| DAPK3 | <50 | <50 |
| DCAMKL1 | <50 | <50 |
| DCAMKL2 | <50 | <50 |
| DCAMKL3 | <50 | <50 |
| **DDR1** | **99** | **100** |
| DDR2 | **100** | 100 |
| DLK | <50 | <50 |
| DMPK | <50 | <50 |
| DMPK2 | <50 | <50 |
| DRAK1 | <50 | 94 |
| DRAK2 | <50 | <50 |
| DYRK1A | <50 | <50 |
| DYRK1B | <50 | <50 |
| DYRK2 | <50 | <50 |
| EGFR | <50 | <50 |
| EGFR(E746-A750del) | <50 | <50 |
| EGFR(G719C) | <50 | <50 |
| EGFR(G719S) | <50 | <50 |
| EGFR(L747-E749del, A750P) | <50 | <50 |
| EGFR(L747-S752del, P753S) | <50 | <50 |
| EGFR(L747-T751del,Sins) | <50 | <50 |
| EGFR(L858R) | <50 | <50 |
| EGFR(L858R,T790M) | <50 | <50 |
| EGFR(L861Q) | <50 | <50 |
| EGFR(S752-I759del) | <50 | <50 |
| EGFR(T790M) | <50 | <50 |
| EIF2AK1 | <50 | <50 |
| EPHA1 | <50 | <50 |
| EPHA2 | <50 | <50 |
| EPHA3 | <50 | <50 |
| EPHA4 | <50 | <50 |
| EPHA5 | <50 | <50 |
| EPHA6 | <50 | <50 |
| EPHA7 | <50 | <50 |
| EPHA8 | <50 | 90 |
| EPHB1 | <50 | <50 |
| EPHB2 | <50 | <50 |
| EPHB3 | <50 | <50 |
| EPHB4 | <50 | <50 |
| EPHB6 | **73** | <50 |
| ERBB2 | <50 | <50 |
| ERBB3 | <50 | <50 |
| ERBB4 | <50 | <50 |
| ERK1 | <50 | <50 |
| ERK2 | <50 | <50 |
| ERK3 | <50 | <50 |
| ERK4 | <50 | <50 |
| ERK5 | <50 | <50 |
| ERK8 | <50 | <50 |
| ERN1 | <50 | <50 |
| FAK | <50 | <50 |
| FER | <50 | <50 |
| FES | <50 | <50 |
| FGFR1 | <50 | <50 |
| FGFR2 | <50 | <50 |
| FGFR3 | <50 | <50 |
| FGFR3(G697C) | <50 | <50 |
| FGFR4 | <50 | <50 |
| FGR | <50 | <50 |
| FLT1 | <50 | <50 |
| FLT3 | <50 | <50 |
| FLT3(D835H) | <50 | <50 |
| FLT3(D835V) | <50 | <50 |
| FLT3(D835Y) | <50 | <50 |
| FLT3(ITD) | <50 | <50 |
| FLT3(ITD,D835V) | <50 | 78 |
| FLT3(ITD,F691L) | <50 | <50 |
| FLT3(K663Q) | <50 | <50 |
| FLT3(N841I) | <50 | <50 |
| FLT3(R834Q) | <50 | <50 |
| FLT3-autoinhibited | <50 | <50 |
| FLT4 | <50 | <50 |
| FRK | <50 | 81 |
| FYN | <50 | <50 |
| GAK | <50 | 81 |
| GCN2(Kin.Dom.2,S808G) | <50 | <50 |
| GRK1 | <50 | <50 |
| GRK2 | <50 | 74 |
| GRK3 | <50 | <50 |
| GRK4 | <50 | <50 |
| GRK7 | <50 | <50 |
| GSK3A | <50 | <50 |
| GSK3B | <50 | <50 |
| HASPIN | <50 | <50 |
| HCK | <50 | 69 |
| HIPK1 | <50 | <50 |
| HIPK2 | <50 | <50 |
| HIPK3 | <50 | <50 |
| HIPK4 | <50 | 92 |
| HPK1 | <50 | <50 |
| HUNK | <50 | <50 |
| ICK | <50 | <50 |
| IGF1R | <50 | <50 |
| IKK-alpha | <50 | <50 |
| IKK-beta | <50 | <50 |
| IKK-epsilon | <50 | <50 |
| INSR | <50 | <50 |
| INSRR | <50 | <50 |
| IRAK1 | <50 | 80 |
| IRAK3 | <50 | <50 |
| IRAK4 | <50 | <50 |
| ITK | <50 | <50 |
| JAK1(JH1domain-catalytic) | <50 | <50 |
| JAK1(JH2domain-pseudokinase) | <50 | 78 |
| JAK2(JH1domain-catalytic) | <50 | <50 |
| JAK3(JH1domain-catalytic) | <50 | <50 |
| JNK1 | <50 | 98 |
| JNK2 | <50 | 82 |
| JNK3 | <50 | 96 |
| KIT | <50 | 100 |
| KIT(A829P) | <50 | 95 |
| KIT(D816H) | <50 | 86 |
| KIT(D816V) | <50 | 89 |
| KIT(L576P) | <50 | 100 |
| KIT(V559D) | <50 | 100 |
| KIT(V559D,T670I) | <50 | 83 |
| KIT(V559D,V654A) | <50 | 99 |
| KIT-autoinhibited | <50 | 74 |
| LATS1 | <50 | <50 |
| LATS2 | <50 | <50 |
| LCK | <50 | 100 |
| LIMK1 | <50 | <50 |
| LIMK2 | <50 | <50 |
| LKB1 | <50 | <50 |
| LOK | **99** | <50 |
| LRRK2 | <50 | <50 |
| LRRK2(G2019S) | <50 | <50 |
| LTK | <50 | <50 |
| LYN | <50 | 90 |
| LZK | <50 | <50 |
| MAK | <50 | <50 |
| MAP3K1 | <50 | <50 |
| MAP3K15 | <50 | <50 |
| MAP3K2 | <50 | <50 |
| MAP3K3 | <50 | <50 |
| MAP3K4 | <50 | <50 |
| MAP4K2 | <50 | <50 |
| MAP4K3 | <50 | <50 |
| MAP4K4 | <50 | <50 |
| MAP4K5 | <50 | <50 |
| MAPKAPK2 | <50 | <50 |
| MAPKAPK5 | <50 | <50 |
| MARK1 | <50 | <50 |
| MARK2 | <50 | <50 |
| MARK3 | <50 | <50 |
| MARK4 | <50 | <50 |
| MAST1 | <50 | <50 |
| MEK1 | <50 | <50 |
| MEK2 | <50 | <50 |
| MEK3 | <50 | <50 |
| MEK4 | <50 | 65 |
| MEK5 | <50 | <50 |
| MEK6 | <50 | <50 |
| MELK | <50 | 85 |
| MERTK | <50 | <50 |
| MET | <50 | <50 |
| MET(M1250T) | <50 | <50 |
| MET(Y1235D) | <50 | <50 |
| MINK | <50 | <50 |
| MKK7 | <50 | <50 |
| MKNK1 | <50 | <50 |
| MKNK2 | <50 | <50 |
| MLCK | <50 | <50 |
| MLK1 | <50 | <50 |
| MLK2 | <50 | <50 |
| MLK3 | <50 | <50 |
| MRCKA | <50 | <50 |
| MRCKB | <50 | <50 |
| MST1 | <50 | <50 |
| MST1R | <50 | <50 |
| MST2 | <50 | <50 |
| MST3 | <50 | <50 |
| MST4 | <50 | <50 |
| MTOR | <50 | <50 |
| MUSK | <50 | <50 |
| MYLK | <50 | <50 |
| MYLK2 | <50 | <50 |
| MYLK4 | <50 | <50 |
| MYO3A | <50 | <50 |
| MYO3B | <50 | <50 |
| NDR1 | <50 | <50 |
| NDR2 | <50 | <50 |
| NEK1 | <50 | <50 |
| NEK10 | <50 | <50 |
| NEK11 | <50 | <50 |
| NEK2 | <50 | <50 |
| NEK3 | <50 | <50 |
| NEK4 | <50 | <50 |
| NEK5 | <50 | <50 |
| NEK6 | <50 | <50 |
| NEK7 | <50 | 65 |
| NEK9 | <50 | <50 |
| NIK | <50 | <50 |
| NIM1 | <50 | <50 |
| NLK | <50 | <50 |
| OSR1 | <50 | <50 |
| p38-alpha | <50 | <50 |
| p38-beta | <50 | <50 |
| p38-delta | <50 | <50 |
| p38-gamma | <50 | <50 |
| PAK1 | <50 | <50 |
| PAK2 | <50 | <50 |
| PAK3 | <50 | <50 |
| PAK4 | <50 | <50 |
| PAK6 | <50 | <50 |
| PAK7 | <50 | <50 |
| PCTK1 | <50 | <50 |
| PCTK2 | <50 | <50 |
| PCTK3 | <50 | <50 |
| PDGFRA | <50 | 100 |
| PDGFRB | <50 | 100 |
| PDPK1 | <50 | <50 |
| PFCDPK1(P.falciparum) | <50 | <50 |
| PFPK5(P.falciparum) | **81** | <50 |
| PFTAIRE2 | <50 | <50 |
| PFTK1 | <50 | <50 |
| PHKG1 | <50 | <50 |
| PHKG2 | <50 | <50 |
| PIK3C2B | <50 | <50 |
| PIK3C2G | <50 | <50 |
| PIK3CA | <50 | <50 |
| PIK3CA(C420R) | <50 | <50 |
| PIK3CA(E542K) | <50 | <50 |
| PIK3CA(E545A) | <50 | <50 |
| PIK3CA(E545K) | <50 | <50 |
| PIK3CA(H1047L) | <50 | <50 |
| PIK3CA(H1047Y) | <50 | <50 |
| PIK3CA(I800L) | <50 | <50 |
| PIK3CA(M1043I) | <50 | <50 |
| PIK3CA(Q546K) | <50 | <50 |
| PIK3CB | <50 | <50 |
| PIK3CD | <50 | <50 |
| PIK3CG | <50 | <50 |
| PIK4CB | <50 | <50 |
| PIKFYVE | <50 | <50 |
| PIM1 | <50 | <50 |
| PIM2 | <50 | <50 |
| PIM3 | <50 | <50 |
| PIP5K1A | <50 | <50 |
| PIP5K1C | <50 | <50 |
| PIP5K2B | <50 | <50 |
| PIP5K2C | <50 | 97 |
| PKAC-alpha | <50 | <50 |
| PKAC-beta | <50 | <50 |
| PKMYT1 | <50 | <50 |
| PKN1 | <50 | <50 |
| PKN2 | <50 | <50 |
| PKNB(M.tuberculosis) | <50 | <50 |
| PLK1 | <50 | <50 |
| PLK2 | <50 | <50 |
| PLK3 | <50 | <50 |
| PLK4 | <50 | <50 |
| PRKCD | <50 | <50 |
| PRKCE | <50 | <50 |
| PRKCH | <50 | <50 |
| PRKCI | <50 | <50 |
| PRKCQ | <50 | <50 |
| PRKD1 | <50 | <50 |
| PRKD2 | <50 | <50 |
| PRKD3 | <50 | <50 |
| PRKG1 | <50 | <50 |
| PRKG2 | <50 | <50 |
| PRKR | <50 | <50 |
| PRKX | <50 | <50 |
| PRP4 | <50 | <50 |
| PYK2 | <50 | <50 |
| QSK | <50 | <50 |
| RAF1 | <50 | 90 |
| RET | <50 | <50 |
| RET(M918T) | <50 | <50 |
| RET(V804L) | <50 | <50 |
| RET(V804M) | <50 | <50 |
| RIOK1 | <50 | <50 |
| RIOK2 | <50 | 66 |
| RIOK3 | <50 | <50 |
| RIPK1 | <50 | <50 |
| RIPK2 | <50 | <50 |
| RIPK4 | <50 | <50 |
| RIPK5 | <50 | <50 |
| ROCK1 | <50 | <50 |
| ROCK2 | <50 | <50 |
| ROS1 | <50 | 67 |
| RPS6KA4(Kin.Dom.1-N-terminal) | <50 | <50 |
| RPS6KA4(Kin.Dom.2-C-terminal) | <50 | <50 |
| RPS6KA5(Kin.Dom.1-N-terminal) | <50 | <50 |
| RPS6KA5(Kin.Dom.2-C-terminal) | <50 | <50 |
| RSK1(Kin.Dom.1-N-terminal) | <50 | <50 |
| RSK1(Kin.Dom.2-C-terminal) | <50 | <50 |
| RSK2(Kin.Dom.1-N-terminal) | <50 | <50 |
| RSK2(Kin.Dom.2-C-terminal) | <50 | <50 |
| RSK3(Kin.Dom.1-N-terminal) | <50 | <50 |
| RSK3(Kin.Dom.2-C-terminal) | <50 | <50 |
| RSK4(Kin.Dom.1-N-terminal) | <50 | <50 |
| RSK4(Kin.Dom.2-C-terminal) | <50 | <50 |
| S6K1 | <50 | <50 |
| SBK1 | <50 | <50 |
| SGK | <50 | <50 |
| SgK110 | <50 | <50 |
| SGK2 | <50 | <50 |
| SGK3 | <50 | <50 |
| SIK | <50 | <50 |
| SIK2 | <50 | <50 |
| SLK | <50 | <50 |
| SNARK | <50 | <50 |
| SNRK | <50 | <50 |
| SRC | <50 | <50 |
| SRMS | <50 | <50 |
| SRPK1 | <50 | <50 |
| SRPK2 | <50 | <50 |
| SRPK3 | <50 | <50 |
| STK16 | <50 | <50 |
| STK33 | <50 | <50 |
| STK35 | <50 | <50 |
| STK36 | <50 | <50 |
| STK39 | <50 | <50 |
| SYK | <50 | <50 |
| TAK1 | <50 | <50 |
| TAOK1 | <50 | <50 |
| TAOK2 | <50 | <50 |
| TAOK3 | <50 | <50 |
| TBK1 | <50 | <50 |
| TEC | <50 | <50 |
| TESK1 | <50 | <50 |
| TGFBR1 | <50 | <50 |
| TGFBR2 | <50 | <50 |
| TIE1 | **70** | <50 |
| TIE2 | <50 | <50 |
| TLK1 | <50 | <50 |
| TLK2 | <50 | <50 |
| TNIK | <50 | <50 |
| TNK1 | <50 | <50 |
| TNK2 | <50 | <50 |
| TNNI3K | <50 | 65 |
| TRKA | <50 | <50 |
| TRKB | <50 | <50 |
| TRKC | <50 | <50 |
| TRPM6 | <50 | <50 |
| TSSK1B | <50 | <50 |
| TSSK3 | <50 | <50 |
| TTK | <50 | <50 |
| TXK | <50 | <50 |
| TYK2(JH1domain-catalytic) | <50 | <50 |
| TYK2(JH2domain-pseudokinase) | <50 | <50 |
| TYRO3 | <50 | <50 |
| ULK1 | <50 | <50 |
| ULK2 | <50 | <50 |
| ULK3 | <50 | <50 |
| VEGFR2 | <50 | <50 |
| VPS34 | <50 | 84 |
| VRK2 | <50 | <50 |
| WEE1 | <50 | <50 |
| WEE2 | <50 | <50 |
| WNK1 | <50 | <50 |
| WNK2 | <50 | <50 |
| WNK3 | <50 | <50 |
| WNK4 | <50 | <50 |
| YANK1 | <50 | <50 |
| YANK2 | <50 | <50 |
| YANK3 | <50 | <50 |
| YES | <50 | <50 |
| YSK1 | <50 | <50 |
| YSK4 | <50 | <50 |
| ZAK | <50 | 73 |
| ZAP70 | <50 | <50 |
