# Supplementary material for: Selective pharmacological inhibition of DDR1 prevents experimentally-induced glomerulonephritis in prevention and therapeutic regime
Source: J Transl Med. 2018 Jun 1;16:148. doi: 10.1186/s12967-018-1524-5 (PMC5984769; doi:10.1186/s12967-018-1524-5)
Supplement: Supplementary file 3 — Additional file 3. Additional materials. [file 12967_2018_1524_MOESM3_ESM.docx]

**Additional Materials**

**Additional Table 1 - DDR1 variants and primers used for identification**

**Additional Table 2 - DDR1i selectivity profile**

Selectivity profile for DDR1i and Imatinib for 451 kinases tested concentration of 1µM. Lower values indicate weaker binding.

**Additional Table 3 - NTS raw fold change signals**

**Additional Table 4 – DDR1 specific MoA genes**

**mRNA expression of the different DDR1 isoforms in healthy and diseased human kidney**

The PCR primers used are described in Additional Table 1. Expression was assessed in sections from normal areas of kidney tissues obtained from surgical resections of kidneys harboring carcinoma, in explanted kidneys from patients with polycystic kidney disease (PKD), and in explanted kidneys with evident histological signs of end stage renal disease (ESRD) (Additional Figure 1A). DDR1 mRNA was expressed in all the three groups to a very similar level and was not significantly modulated by disease, either ADPKD or ESRD. Out of the six DDR1 mRNA isoforms, DDR1 v2, coding for the DDR1b isoform, appeared to be the major type of DDR1 expressed in all conditions (Suppl Figure 1).

**Specificity and sensitivity of the monoclonal anti-human DDR1 antibody**

For IHC analyses, we generated an in-house rabbit monoclonal antibody directed against the extracellular domain of human DDR1, referred as DDR1-Rb819. Analyses of DDR1-Rb819 revealed that it is unable to recognize DDR1 in western blot analyses, but specifically identifies DDR1 in fixed tissues using IHC. Indeed, IHC analyses using DDR1-Rb819 on two different human pancreatic cancer cell lines with defined DDR1 expression, as determined by PCR analyses (not shown) and western blot analyses (Additional figure 2), using the commercially available DDR1antibody D1G6, revealed specifc immunoreactivity with DDR1-Rb819 antibody in DDR1-expressing CFPAC-1 cells but not in DDR1-negative PANC-1 cells (Additional figure 2). The DDR1 staining in CFPAC-1 cells with DDR1-Rb819 was readily detected at the cell membrane, consistent with DDR1 being a membrane-tethered RTK. Immunostaining using a control rabbit IgG was negative. Further experiments on NMuMG cell line (ATCC, CRL-1636™) overexpressing the murine DDR1 protein were negative, demonstrating restricted reactivity to human DDR1 (data not shown).

**Inhibition profile analysis of Roche-Chugai DDR1i**

As shown in Additional Figure 7, inhibition profile analyses showed that at a concentration of 1 μM against a panel of 451 kinases (including 59 mutant kinases), the Roche-Chugai DDR1i showed a remarkable target selectivity with selectivity scores of S_1_(65) of 0.005 and S_1_(90) of 0.0026, respectively, with the only target inhibited greater than 50% at this concentration being DDR2 (Additional Table 2). Thus, Roche-Chugai DDR1i is a highly selective DDR1 kinase inhibitor. Roche-Chugai DDR1i was also evaluated for pharmacokinetics and tissue availability in mice to determine the optimal inhibitor doses for *in vivo* experiments, aiming DDR1-phosporylation inhibition >90% in average over 24 hours (Additional Figure 4). These analyses guided the doses used in this study. Roche-Chugai DDR1i exhibited negligible toxicity at the utilized doses. With this information in hand, we set to examine the effect of Roche-Chugai DDR1i administration in the development and progression of NTS-induced crescentic GN in mice.

**Selectivity profile of DDR1i and Imatinib**

Consistently with previously published data by Day et *al*.[^20^](#_ENREF_24), our *in vitro* analyses confirmed the ability of Imatinib to bind and inhibit DDR1 phosphorylation. The kinetic inhibition data showed that Imatinib inhibited DDR1 with similar potency than Roche-Chugai DDR1i. Specifically, both Imatinib and Roche-Chugai DDR1i strongly inhibited DDR1 kinase activity with IC_50_ values of 0.021 μM and 0.010 μM, respectively. Moreover, in an ELISA-based cellular assay both compounds potently inhibited receptor phosphorylation with IC_50_ values of 0.038 μM (Imatinib) and 0.014 μM (DDR1i). However, Imatinib and Roche-Chugai DDR1i displayed a very different kinase selectivity profile, as assessed in an active-site-dependent competition binding assay (DiscoveRx Corporation, San Diego, USA DiscoverX scanMAX℠ Kinase Assay Panel) (Additional Figure 8). Indeed, at a 1 μM concentration, the selectivity of Roche DDRi was significantly higher than that of Imatinib, which showed selectivity scores S_1_(65) and S_1_(90) of 0.036 and 0.020, respectively (Additional Figure 5).

**DDR1 inhibition decreases PEC pro-inflammatory phenotype *in vitro***

In order to validate these results *in vitro*, we analyzed the impact of DDR1 inhibition on cellular signalling using mouse primary PECs[^15^](#_ENREF_18). Both DDR1 phosphorylation and mRNA expression of some inflammation/fibrosis proteins were measured. Pre-treatment of PECs for 1h with DDR1i followed by treatment with type I collagen for 6h blocked DDR1 phosphorylation in a dose dependent manner (Additional Figure 6A). Treatment with type I collagen alone induced a significant increase of the matrix metalloproteinases Mmp14 and Mmp2, complement component C3 and vascular cell adhesion molecule 1 VCAM1 mRNA (Additional Figure 6B-E). Pre-treatement of the cells with DDR1i caused a dose-dependent inhibition of expression of these genes.
